# Deciphering the Effect of Microstructural Modification in Sodium Alginate-Based Solid Polymer Electrolyte by Unlike Anions

**DOI:** 10.1021/acsomega.3c05094

**Published:** 2023-11-13

**Authors:** Supriya K. Shetty, Ismayil, Ikhwan Syafiq Mohd Noor, Sudhakar Narahari Yethadka, Pradeep Nayak

**Affiliations:** † Department of Physics, Manipal Institute of Technology, Manipal Academy of Higher Education, Manipal 576104, Karnataka, India; ‡ Physics Division, Centre of Foundation Studies for Agricultural Science, Universiti Putra Malaysia, 43400 Serdang, Selangor Darul Ehsan, Malaysia; § Department of Chemistry, Manipal Institute of Technology, Manipal Academy of Higher Education, Manipal 576104, Karnataka, India

## Abstract

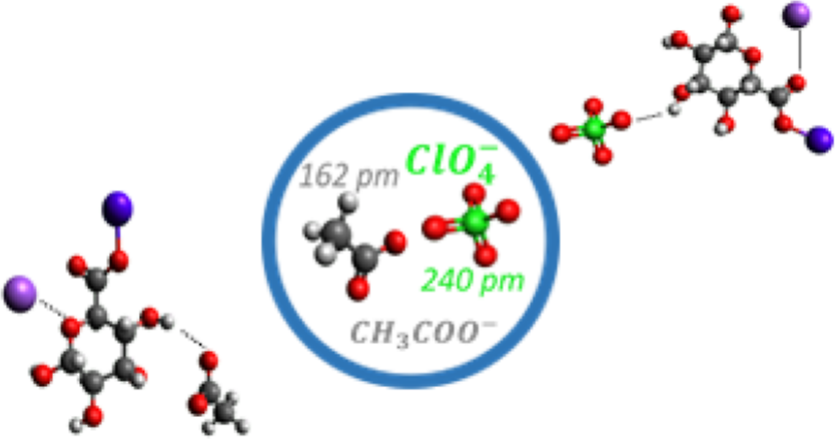

Microstructure modification in sodium alginate (NaAlg)-based solid polymer electrolytes by the perchlorate (ClO_4_^–^) and acetate (CH_3_COO^–^) anions of sodium salts has been reported. ClO_4_^–^ participates in the structure-breaking effect via inter/intramolecular hydrogen bond breaking, while CH_3_COO^–^ changes the amorphous phase, as evident from X-ray diffraction studies. The larger size and negative charge delocalization of ClO_4_^–^ have a plasticizing effect, resulting in a lower glass transition temperature (*T*_g_) compared to CH_3_COO^–^. Decomposition temperature is strongly dependent on the type of anion. Scanning electron microscopy images showed divergent modifications in the surface morphology in both electrolyte systems, with variations in salt content. The mechanical properties of the NaAlg–NaClO_4_ electrolyte systems are better than those of the NaAlg–CH_3_ COONa system, indicating weak interactions in the latter. Although most of the studies focus on the cation influence on conductivity, the interaction of the anion and its size certainly have an influence on the properties of solid polymer electrolytes, which will be of interest in the near future for sodium ion-based electrolytes in energy storage devices.

## 1 Introduction

Rechargeable batteries are a new generation of electrical power sources.^1^ Among the various types of rechargeable batteries, lithium-ion batteries (LIBs) are gaining global attention, have been ruling the nonrechargeable battery market for the past 40 years, and are expected to grow at a compound annual growth rate of 12.3% from 2021 to 2023. However, LIBs easily tend to overheat and can break down at high voltages, leading to safety issues. As such, sodium-ion batteries (SIBs) are presented as an alternative to LIBs due to their high sustainability, affordability, safety, and scalability.^2^ Sodium is abundant in the earth’s crust, mined from trona, and sodium ash and its presence in seawater make it cheaper than lithium. The wider operating temperature range of SIB (−70 to 100 °C) compared to LIB (−20 to 60 °C) provides better safety.^3,4^ In SIB, aluminum (Al) is used as a current collector (Na does not react with Al). This makes the SIBs cheaper and lighter,^5^ and they also can be discharged to 0 V (which is not possible in LIBs) due to the use of Al, which helps facilitate charge transport. Since research on SIBs is still in its infancy, the energy density of SIBs is still low compared to LIBs.^6^ Therefore, the need for rigorous work toward the development of SIBs to meet the expected demand is to be intensified.

The performance of the battery depends on the fabricated electrodes and the electrolyte. The electrolyte is responsible for the rapid transport of ions between the electrodes during the intercalation–deintercalation process in a rechargeable battery. Therefore, attention to electrolyte development with high performance should be emphasized. Polymer electrolytes have emerged as a potential candidate for next-generation wearable energy storage devices due to their excellent flexibility. In this regard, solid polymer electrolytes are suitable and advantageous because they have good thermal stability, a wider operating voltage, and better safety compared to liquid electrolytes. However, solid polymer electrolytes have low ionic conductivity and cyclability that need to be improved.^7,8^ Realizing the growing environmental issues realized, biomass-derived biopolymers and electrode materials are of great importance. Biopolymer-based electrolytes have exhibited good capabilities in energy storage and conversion devices such as zinc-ion batteries and lithium-ion batteries.^9^ Although synthetic polymers such as poly(ethylene oxide), poly(vinylpyrrolidone), poly(methyl methacrylate), poly(vinylidene fluoride), poly(vinyl alcohol), and poly(acrylonitrile) are extensively explored as polymer hosts,^10^ in recent years, biodegradable polymers have attracted the attention of researchers as an alternative to synthetic polymers due to their low cost, rich resources, nontoxicity, degradability, and biocompatibility.^11^ Polysaccharides such as cellulose, carboxymethyl cellulose, methylcellulose, hydroxyethyl cellulose, chitin, chitosan, potato starch, wheat starch, carrageen, xylan, dextran, pectin, and sodium alginate are natural polymers used as host polymers in polymer electrolytes.^12,13^ These natural polymers have great potential to be used in energy storage applications.

Sodium alginate (NaAlg) is a water-soluble polysaccharide with abundant surface functional groups such as free hydroxyl and carbonyl groups on its polymer matrix. Monomers of mannuronic acid (M) and guluronic acid (G) are the building blocks of alginate. It is a good candidate as a polymer host in polymer electrolytes because of its relative dielectric and has also emerged as one of the best eco-friendly water-soluble binders in electrode preparation.^14^ Several reports are available with NaAlg as a polymer host for energy storage applications. Diana et al., doped sodium iodide and sodium thiocyanate into the NaAlg matrix and achieved conductivities of 10^–2^ S cm^–1^^15^ and 1.22 × 10^–2^ S cm^–1^,^16^ respectively, for 70 wt % of salt. Fuzlin et al. attained the highest conductivity of 7.46 × 10^–5^ S cm^–1^ for 15 wt % of LiBr salt in the alginate matrix.^17^ A solid polymer electrolyte is produced by dissolving the appropriate salt in a polymer matrix. The solubility of salts in the polymer matrix depends on the dielectric constant of the polymer, which is determined by the availability of the functional groups in the polymer host to dissociate the salt. A lack of guidelines has been observed in selecting suitable salts as complexes,^18^ and thus this research gap needs to be addressed. From the list of sodium salts, NaClO_4_, NaI, NaNO_3_, NaPF_6_, NaBF_4_, NaBOB, NaOTf, and CH_3_COONa, it is difficult to choose the appropriate salt since each of them has its own advantages and disadvantages. Well-defined criteria for selecting the appropriate salt are necessary based on the application.

A salt must exhibit good chemical, electrochemical, and thermal stability to be the right choice in the production of polymer electrolytes. Microstructure modification in the solid polymer electrolyte is always associated with the coordination of the cation with the polymer chains, and the binding of an anion with the polymer matrix is not well addressed. The effect of anions on the properties of solid polymer electrolytes (SPEs) and the ion transport mechanism have still not been widely studied. It is important to know whether the salt used can achieve the properties required by SPEs. Based on our literature survey, there are very few reports on the influence of anion size on the microstructural properties of SPEs. A fundamental understanding of how the anion size, charge delocalization, charge density, anion–cation strength, and ion–dipolar interactions affect the ionic conductivity and chemical/electrochemical properties of SPE is still unclear and needs to be rectified. The role of functional groups attached to polymer chains in dissociating ions and their influence on ionic conductivity are still poorly understood. In this report, sodium perchlorate (NaClO_4_) (ionic radius = 240 pm) and sodium acetate (CH_3_COONa) (ionic radius = 162 pm)^19^ are chosen as complex salts to prepare an SPE based on NaAlg polymer to understand the effect of the anion size of the salt on the properties of SPE. The hypothesis is that the size and nature of anions will have an impact on the microstructural properties of polymer electrolytes and will play a crucial role in achieving the required properties for a solid polymer electrolyte.

## 2 Results and Discussion

### 2.1 Fourier Transform Infrared and Raman Spectroscopy

**Figure 1:**
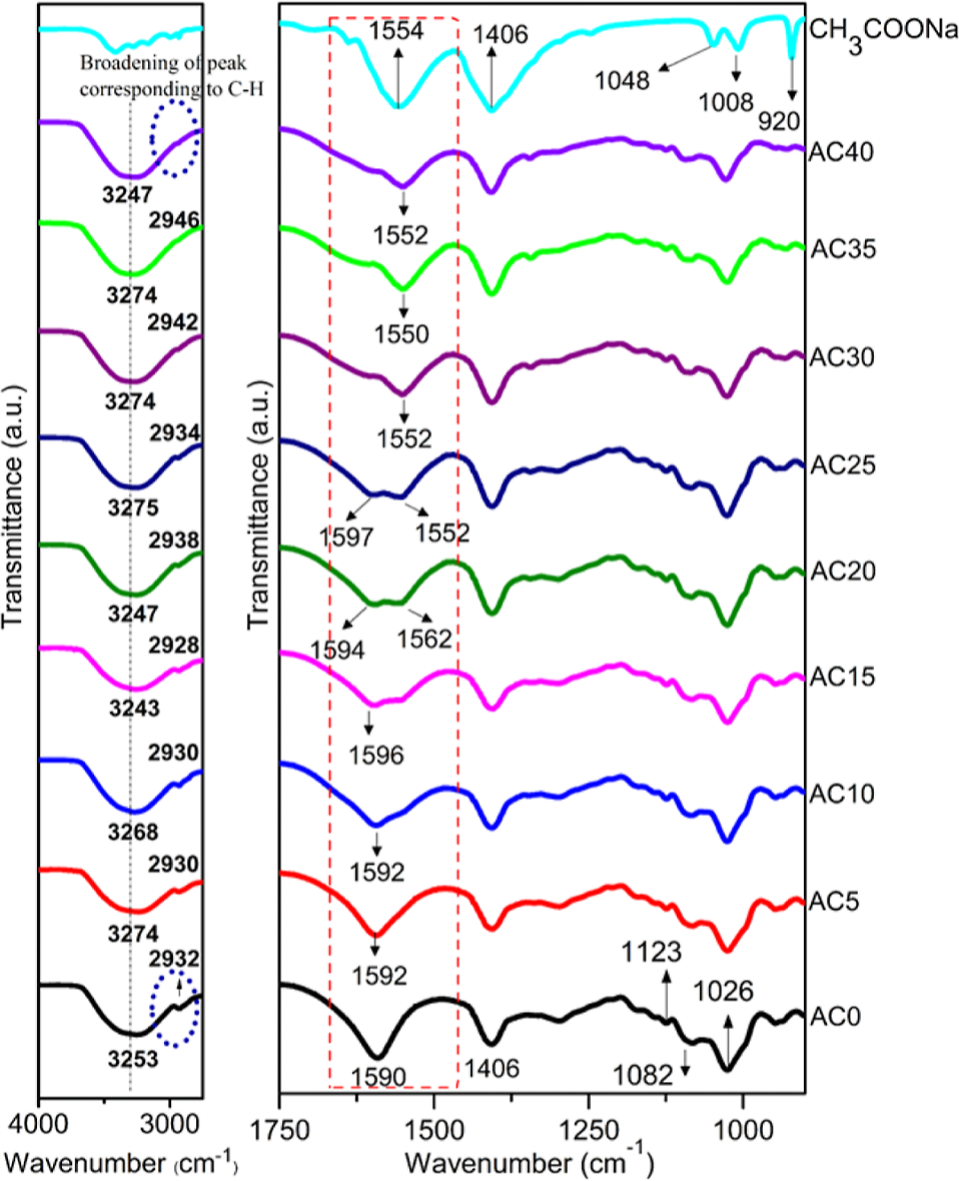
FTIR spectra of NaAlg, CH_3_COONa, and NaAlg–CH_3_COONa SPEs.

The Fourier transform infrared (FTIR) spectrum of (CH_3_COONa) salt and NaAlg-based SPE with different concentrations of CH_3_COONa salt composition is shown in Figure 1. NaAlg exhibited a broad band in the wavenumber between 3000 and 3700 cm^–1^ corresponding to stretching vibrations (*v*_s_) of the hydrogen-bonded –OH group. A weak band at 2932 cm^–1^ is assigned to the asymmetric stretching vibration (*v*_as_) of C–H.^20,21^ A strong-intense band followed by a medium-intense peak at 1590 and 1406 cm^–1^, are referred to ν_as_ and ν_s_ stretching vibrations of the carboxylate group (COO^–^), respectively.^22^ A band at 1297 cm^–1^ is assigned to C–C–H and C–O–H deformation of the pyranose ring (skeletal vibration).^23,24^ Several bands observed in the wavenumber region between 1200 and 950 cm^–1^, the so-called fingerprint region of carbohydrates, correspond to C–O and C–C stretching modes, respectively. The bands observed at 1123, 1082, and 1026 cm^–1^ respectively refer to C–C and C–O stretching vibrations of the pyranose ring and C–O–C stretching of glycosidic bonds, which is commonly observed in polysaccharides, resulting from the coupling of different vibration modes.^23,25−27^

In Figure 1, the FTIR spectrum of NaAlg–CH_3_COONa shows a change in the absorption band of the polymer functional group due to salt incorporation. The formation of ion complexes with the polar groups of the polymer affects the vibrational spectrum, and the contribution of these ions to the ionic conductivity is determined by the nature of the ion–polymer interaction. The Na^+^ ion interacts primarily with the oxygen of the hydroxyl group (–OH) and carboxylate group (COO^–^) and its interaction with the polymer is difficult to probe (significant for observation).^28^ In Figure 1, sodium acetate salt has two prominent peaks at 1554 and 1406 cm^–1^, which, respectively, correspond to ν_s_ and ν_as_ stretching of the carboxylate group of the acetate anion. A peak at 1048 and 1008 cm^–1^ corresponds to out-of-plane and in-plane –CH_3_ rocking of the methyl group, respectively. A band at 920 cm^–1^ corresponds to C–C stretching.^29,30^

The formation, disappearance, and change in position as well as the shape of the FTIR peaks observed in the electrolyte system prove that the complexation between NaAlg and p CH_3_COONa has occurred. The broadening of the peak corresponding to C–H vibration (2929 cm^–1^, at higher salt concentrations) is attributed to the formation of a hydrogen bond between the oxygen of CH_3_COO^–^ anion and the hydrogen of the C–H group. Overlapping of the CH_3_COO^–^anion at 1554 cm^–1^ with the ν_as_(COO^–^) of NaAlg at 1590 cm^–1^ has resulted in variation of shape, position, and intensity of the band corresponding to ν_as_(COO^–^). In Figure 1, a single peak at 1590 cm^–1^ is resolved into two peaks after doping. The peak at (∼1554 cm^–1^) corresponds to ν_as_(COO^–^) of the acetate ion, and the intensity of this peak increases with increasing salt concentration. While the peak observed at ∼1590 cm^–1^ corresponds to ν_as_(COO^–^) of the polymer. The intensity of the band corresponding to ν_as_(COO^–^) of polymer decreased with an increase in salt concentration and is due to a decrease in their availability. A significant variation in asymmetric stretching of the COO^–^ group was observed due to the difference in wavenumber corresponding to polymer (∼1590 cm^–1^) and anion (∼1554 cm^–1^), and in the case of symmetric stretching of the COO^–^ group, a single peak was observed due to the similarity in wavenumber corresponding to that of polymer (∼1406 cm^–1^) and anion (∼1406 cm^–1^).

**Figure 2:**
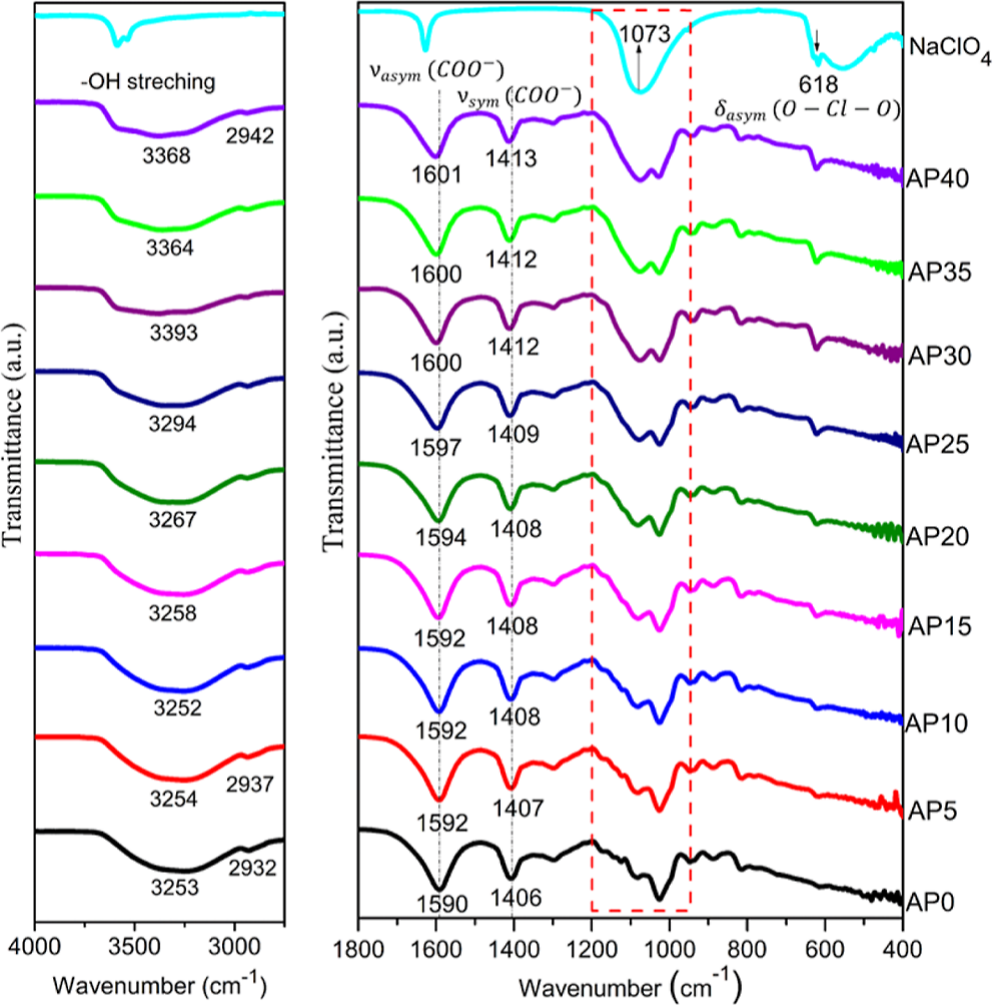
FTIR spectra of NaAlg, NaClO_4_, and NaAlg–NaClO_4_ SPEs.

**Figure 3:**
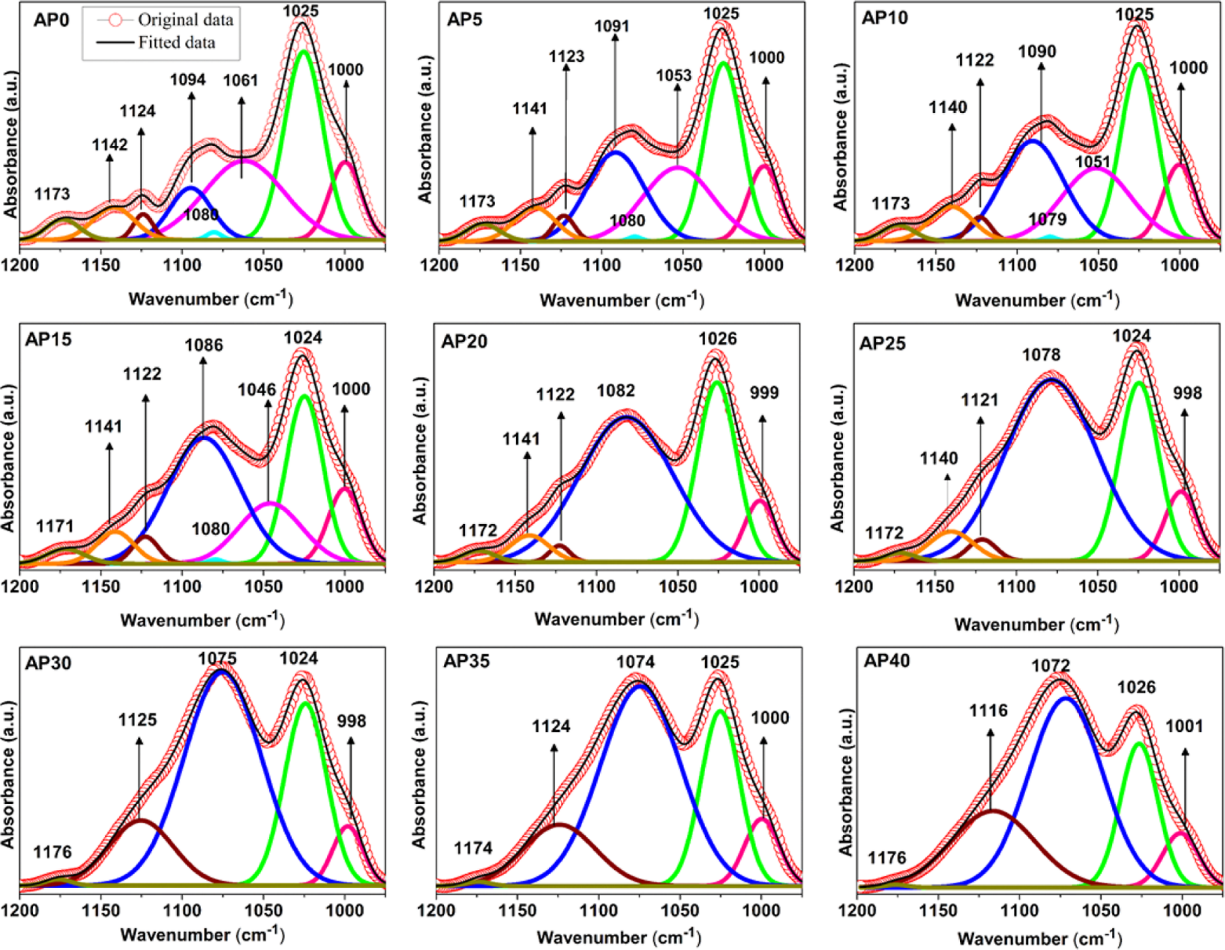
FTIR deconvolution of NaAlg with different weight percentages of sodium perchlorate salt in the wavenumber region between 1200 and 975 cm^–1^.

**Figure 4:**
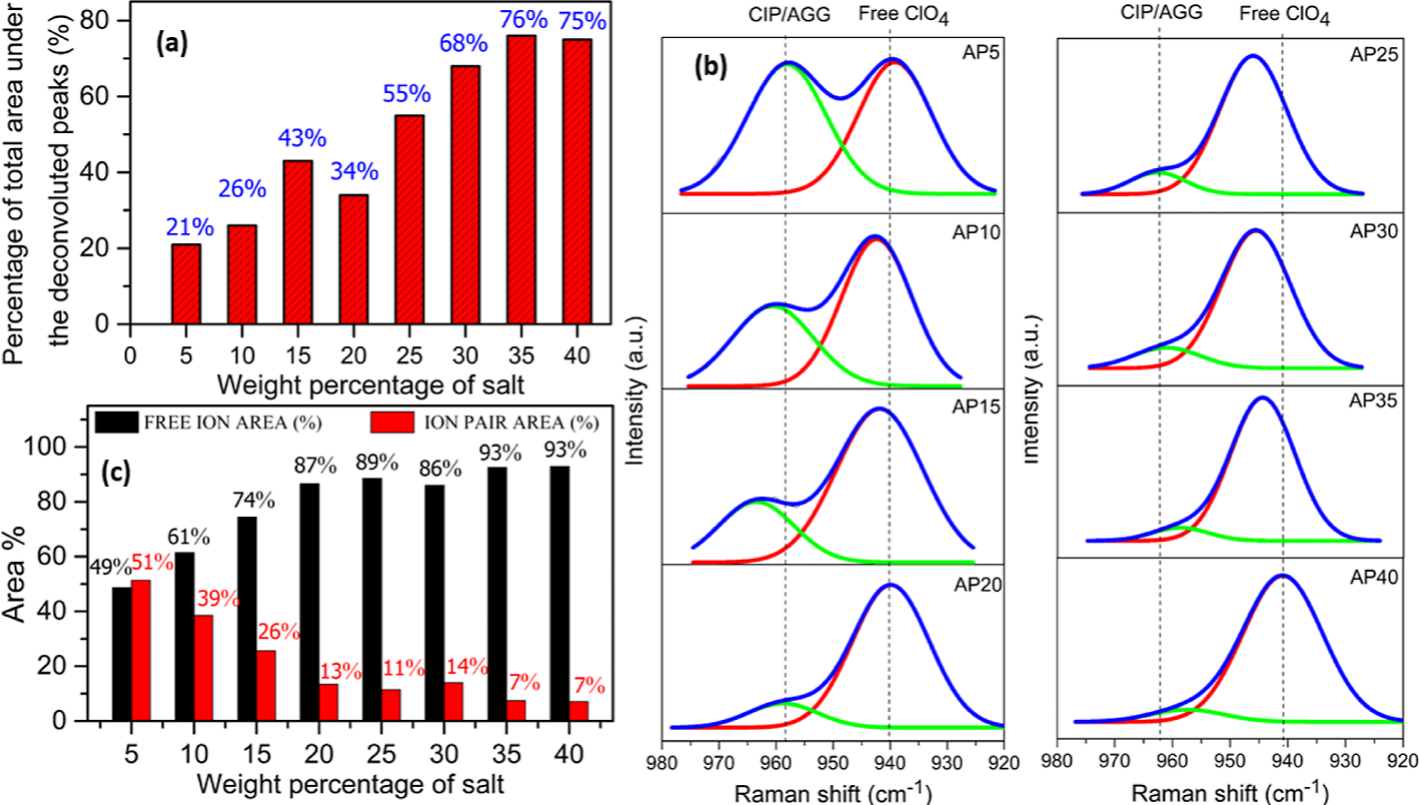
(a) Variation of peak area under the ether region against salt concentration for the NaAlg–NaClO_4_ electrolyte system, (b) deconvoluted Raman spectra of the NaAlg–NaClO_4_ electrolyte system in the range between 920 and 980 cm^–1^, and (c) plot of free ion area and ion pair area against salt concentration.

Figure 2 shows the FTIR spectra of the NaClO_4_ salt and NaAlg–NaClO_4_ SPEs. Free perchlorate anion refers to tetrahedral symmetry (*T*_d_) exhibiting four fundamental modes of vibration (*A*_1_ + *E* + 2*F*_2_), in which modes of vibration ν_1_(*A*_1_) at 933 cm^–1^ (symmetrical stretching), ν_2_(*E*) at 462 cm^–1^ (symmetrical bending), ν_3_(*F*_2_) at 1113 cm^–1^ (asymmetrical stretching), and ν_4_(*F*_2_) at 629 cm^–1^ (asymmetrical bending) are all Raman active. Modes of vibration ν_3_ and ν_4_ are IR active.^31,32^ In this case, the free ClO_4_^–^ anion exhibits only two bands in the IR spectra (Figure 2). A strong and broad IR peak at 1073 cm^–1^ is assigned to asymmetric Cl–O stretching of the ClO_4_^–^ anion. The peak at 618 cm^–1^ is assigned to asymmetric bending (δ_as_(O–Cl–O)). A prominent variation in the band shape was observed in the wavenumber region between 1200 and 950 cm^–1^. This is due to the occurrence of a perchlorate ion peak (1073 cm^–1^) in this region. Thus, FTIR deconvolution was performed to evaluate the overlapping peaks forming the superposition spectrum in the corresponding wavenumber region, which is presented in Figure 3. The absorbance peaks after baseline correction were fitted to the Gaussian function, and the area of the deconvoluted bands was calculated. Overlapping of the perchlorate ion peak with the C–O and C–C stretching vibration bands was observed using the deconvolution technique. Deconvolution of the NaAlg spectrum exhibited eight bands at 1173, 1142, 1124, 1094, 1080, 1061, 1025, and 1000 cm^–1^, which respectively correspond to C–O and C–C stretching modes. The total area of the deconvoluted peaks between the wavenumber regions of 1200 and 975 cm^–1^ increases with increasing salt concentration, indicating an increase in the dissociation of salt into free ions. Thereafter, it reached saturation at a higher salt concentration [in Figure 4a, the area is plotted in percentage with respect to the area of the pristine (AP0)] due to the nonavailability of the polar groups in the polymer matrix to dissociate the salt, which can lead to the formation of salt agglomeration/salt aggregation. (The shift in the band due to the interaction of ions with the polymer host is tabulated in Tables S2 and S3).

To understand the type of interaction of ions with the –OH group, FTIR deconvolution was carried out in the region between 3700 and 3000 cm^–1^ for both electrolyte systems of NaAlg–NaClO_4_ and NaAlg–CH_3_COONa (refer Figures S1 and S2). The deconvolution of the pristine state in this region has resulted in two peaks. The peak ∼3200 cm^–1^ corresponds to the stretching vibration of the intermolecular hydrogen-bonded –OH group. The peak at ∼3400 cm^–1^ is attributed to the intramolecular hydrogen-bonded –OH group. No peak is observed due to the free –OH group,^33,34^ indicating that all hydroxyl groups are hydrogen-bonded. Since the band related to free hydroxy stretching falls at the higher wavenumber side (∼3600 cm^–1^), the deconvoluted peaks are attributed to the intra- and inter-molecular stretching of the –OH group.^34,35^ A new shoulder peak was observed at the higher end of the wavenumber region between 3700 and 3000 cm^–1^ in the case of the NaAlg–NaClO_4_ electrolyte system. As the concentration of NaClO_4_ salt increases in the polymer matrix, a significant increase in the intensity as well as a shift to a high wavenumber in the newly formed shoulder peak were identified. A new shoulder peak appeared in the doped complexed polymer system at the higher wavenumber region, which is attributed to the interaction of ions with –OH. The anions have the ability to form the hydrogen bond with –CH, –OH, and –NH acidic sites of polymer.^36−39^ Knowledge of the anion interaction with the polymer host and cation is important in designing optimized polymer electrolytes for energy storage applications. The cation, as well as the anion, can influence the –OH stretching, where the Na^+^ cation can interact with the oxygen atom of the polymer functional group and the ClO_4_^–^ anion coordinates with the hydrogen atom of –OH The interactions Na^+^...OH^40,41^ and –OH...ClO_4_^–^^39,42^ can exhibit a peak in the –OH wavenumber region. The cations form weak hydrogen bonds compared with those of anion,^43^ and therefore, the new peak observed can be assigned to the –OH...ClO_4_^–^ interaction.

The intensity of the newly formed peak increases with the increase in the NaClO_4_ salt concentration. This can be attributed to the breaking of the hydrogen bonds by ClO_4_^–^ anions, which is known as the “structure-breaking effect”. In the case of the NaAlg–CH_3_COONa system, no shoulder peak is formed, and the deconvoluted spectra (Figure S2) have only two peaks that correspond to intermolecular and intramolecular hydrogen-bonded –OH group. Even though the interaction of Na^+^...OH is possible, no peak corresponding to this interaction has been observed, indicating a weak interaction of Na^+^with the –OH group.

To understand the presence of free ions and ion pairs in the NaAlg–NaClO_4_ system, which cannot be quantitatively evaluated in the IR region between 1200 and 950 cm^–1^ due to the overlap of the perchlorate anion peak with the band corresponding to C–O–C stretching (consisting of several components). Thus, Raman spectra of the NaAlg–NaClO_4_ complex in the wavenumber region between 1600 and 200 cm^–1^ have been considered since perchlorate anion exhibits a strong and sharp Raman active peak at 954 cm^–1^, as depicted in Figure 4b. A splitting of the symmetric band of the polymer at 957 cm^–1^ into two bands in the NaAlg–NaClO_4_ complex in the wavenumber region between 920 and 980 cm^–1^ has been observed. A new band formed at the lower wavenumber side is assigned to the free ClO_4_^–^anion (940 cm^–1^). The intensity of this band increases with an increase in salt concentration in the polymer matrix. The contact ion pair (CIP/AGG (ion aggregate)) of Na^+^...ClO_4_^–^ is observed at a higher wavenumber of 958 cm^–1^.^44^ Raman deconvoluted spectra in the region between 920 and 980 cm^–1^ are depicted in Figure 4b. Information such as the area under the free ion and contact ion peak obtained from the deconvoluted FTIR was then used to evaluate the transport parameters of SPEs as per the technique explained in the reported article.^45^ The percentage of free ions increased and reached a maximum for higher salt concentrations due to the nonavailability of the same functional group to further dissociate the salt, as observed in Figure 4c. For the AP30 sample, even though there is a decrease in free ion percentage, that does not mean the formation of salt aggregates since the peak intensity in the region for 3700 and 3000 cm^–1^ is high compared to that of AP35, which indicates the dissociation of ions via the –OH group. For the NaAlg–NaClO_4_ system, the percentage of free ions has increased with an increase in salt concentration, indicating the dissociation of ions by the functional group available in the matrix.

### 2.2 X-Ray Diffraction

Investigation into the influence of different anion species on the microstructure as well as on the performance of the polymer electrolytes has not been extensively studied. Therefore, this work quantitatively evaluates the impact of two types of sodium salts with anion species ClO_4_^–^ (strong acid anion) and CH_3_COO^–^ (strong basic anion) on the electrolyte crystallinity. The X-Ray diffraction (XRD) pattern of NaAlg (Figure S3) shows the low crystalline nature of the sample, with a crystalline peak at 2θ = 13° corresponding to the hydrogen bonding among the hydroxyl groups (intra/intermolecular interaction).^46−48^ The NaAlg polymer film exhibits two broad peaks at 2θ = 23° and 2θ = 36°, attributed to two amorphous characteristics. The microstructure of a polymer defines its crystallinity. The factors that will bring about a change in the crystallinity of the electrolyte are the interaction of electron-donar groups of the polymer host with the sodium cation, the type of anion, and its interaction with the polymer chain.^49^ To understand the factors that influence the crystallinity of the polymer electrolyte, the degree of crystallinity (χ_c_) was evaluated, as reported in our earlier work.^50^ In the case of the NaAlg–NaClO_4_ system, the peak intensity at 2θ = 13° (corresponding to the crystalline characteristic) is observed to decrease significantly with increasing salt concentration due to the interaction of the ClO_4_^–^ anion with the hydroxyl group^51^ (Figure S4), which has disrupted the inter- and intramolecular hydrogen bonds of the polymer and thus resulted in a decrease in the peak intensity (disrupting the crystalline phase) of the peak at 2θ = 13°. In the case of the NaAlg–CH_3_COONa electrolyte system, the interaction of the CH_3_COO^–^ anion with the –OH group is very weak (Figure S5), and therefore, the peak intensity observed at 2θ = 13° does not change significantly compared to the NaAlg–NaClO_4_ electrolyte system (interaction of the ClO_4_^–^ anion with the –OH group is stronger than that of the CH_3_COO^–^ group). Even though the Na^+^ cation has interacted with the oxygen of the –OH group in both cases, the interaction of the cation with the polymer active site has not significantly affected χ_c_. The interaction of the polymer with the anion is completely different from that of the cation since the strength of complex formation varies according to the type of ion. The variation of χ_c_ in both systems is tabulated in Table S1, and it is difficult to explain the influence of anion type on crystallinity due to comparable variation in both systems. In the case of NaAlg–NaClO_4_, χ_c_ varied due to disruption of hydrogen bonds, and in the case of NaAlg–CH_3_COONa, variation in χ_c_ was observed due to enhancement in the amorphous content in the amorphous region (as illustrated in the graphical abstract). The structure of the polymer is affected predominantly by the type of anion rather than the cation, as per this study. The role of cation as well as anion in the structural change in the polymer electrolyte needs to be addressed.

### 2.3 Differential Scanning Calorimetry

**Figure 5:**
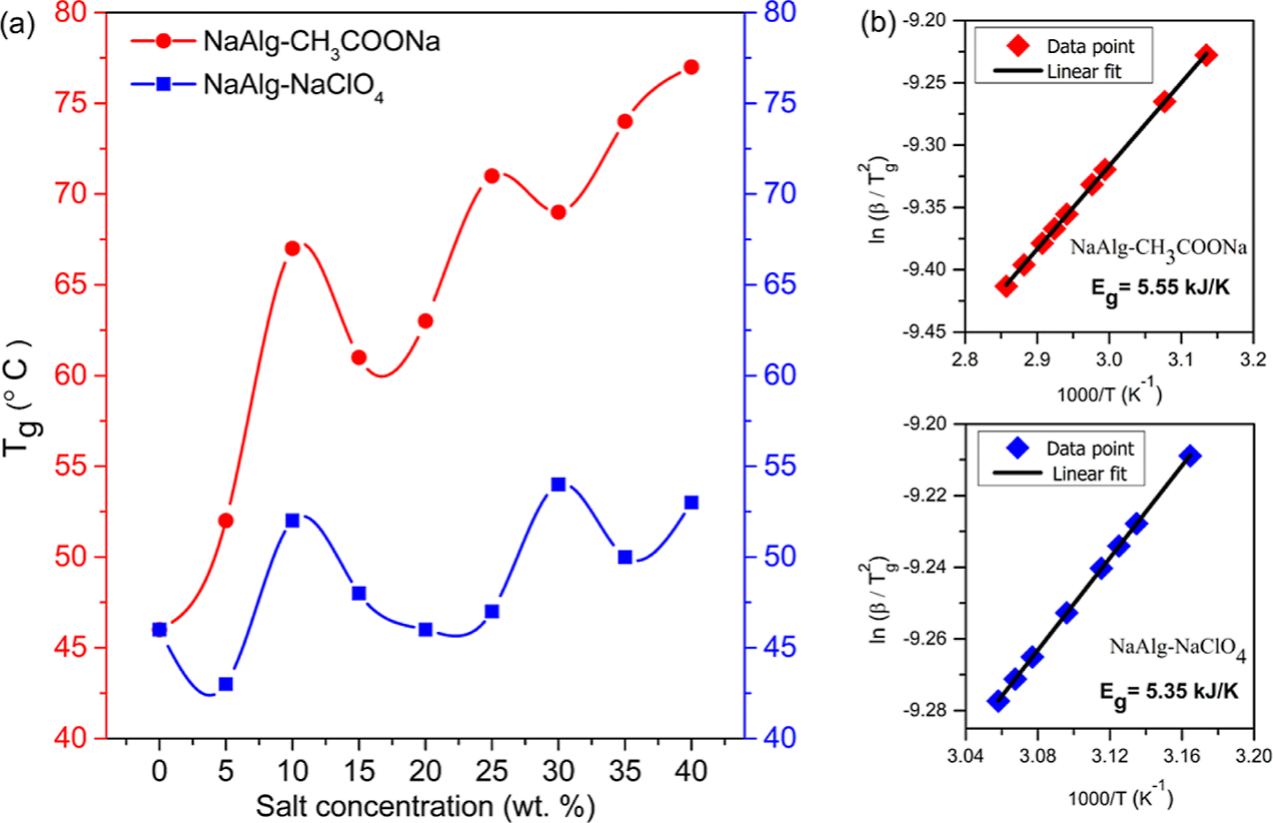
(a) Variation of glass transition temperature (*T*_g_) with salt concentration for the NaAlg–CH_3_COONa and NaAlg–NaClO_4_ electrolyte system, (b) Kissinger’s plot of the *T*_g_ to evaluate the activation energy.

Differential scanning calorimetry (DSC) thermograms of the NaAlg–NaClO_4_ and NaAlg–CH_3_COONa complexed system is depicted in Figure S6. Glass transition temperature (*T*_g_) as a function of the salt concentration for the NaAlg–CH_3_COONa and NaAlg–NaClO_4_ polymer electrolyte systems is depicted in Figure 5a. The *T*_g_ of NaAlg–CH_3_COONa is higher than that of NaAlg–NaClO_4_ at the same salt concentration. The increased *T*_g_ values compared to those of pristine systems indicate that a molecular complexed network has been formed because of ion–dipole interactions. The formation of a physical transient cross-link between the polymer matrix due to the interaction of the polymer chain with Na^+^ cation or Na^+^ anion Na^+^ bridges resulted in the immobilization of the polymer chain segments. A nonlinear increase in *T*_g_ with salt concentration has been observed, indicating the complexity of the microstructure. The discrepancy in *T*_g_ in both systems has arisen from the distinct anions and not due to cation binding, indicating the influence of anion type on the polymer chain mobility.^52^ The *T*_g_ of the polymer electrolyte is determined by the bond rotation and packing of the polymer chains, which depend on the ion interaction with the polymer backbone.^53^ Perchlorate anion (anion radius: 240 pm) is larger than the CH_3_COO^–^ anion (anion radius: 162 pm) and prevents dense packing of the polymer segments, suggesting a strong plasticizing effect of ClO_4_^–^.^54^ The solvation of both anions in the polymer matrix is completely different, and this may be one of the factors that influence the *T*_g_ (since structural modification brought about by the anions is completely different as per XRD). The larger size and high degree of negative charge delocalization of the ClO_4_^–^ anion compared to the CH_3_COO^–^ anion have resulted in a stronger plasticizing effect due to better interaction with the polymer chain, and thus, the *T*_g_ of NaAlg–NaClO_4_ is less than that of the NaAlg–CH_3_COONa electrolyte system.^55^ The Coulombic interaction of the smaller anion (which displays the highest charge density) with the cation is stronger, thus the formation of Na^+^ CH_3_COO^–^ Na^+^ bridges that may enhance the rigidity of the polymer chains due to stronger polymer–salt interaction. The DSC curve of the NaAlg–NaClO_4_ system is broader than that of the NaAlg–CH_3_COONa, concluding the different polymer chain dynamics occurring in both systems (Figure S6). NaAlg has hydrophilic groups such as hydroxyl and carboxyl and therefore can have strong or weak interactions with water molecules, thus influencing its thermal properties. Moisture in the sample can lower the *T*_g_,^52^ but as per the TGA plot (Figures S7 and S8), both systems are prone to absorb moisture, thus concluding that the influence of anion size on *T*_g_ is more prominent over the residual solvent.

Kissinger’s relation,^56^, where *T*_g_ is the glass transition temperature, *R* is the universal gas constant (8.3145 JK^–1^ mol^–1^), *A* is the pre-exponential factor, β is the heating rate (10 K min^–1^), and *E*_g_ is the activation energy of structural relaxation associated with *T*_g_*A* plot of ln(β/g^2^) against 1000/*T*_g_ gives a linear curve, as shown in Figure 5b. The slope of the plot gives information on the activation energy, and it was observed that *E*_g_(NaAlg–CH_3_COONa) > *E*_g_(NaAlg–NaClO_4_) system indicates that less thermal energy is required for structural relaxation of polymer chains in the (NaAlg–NaClO_4_) system due to the flexibility of polymer chains and therefore restricts the diffusivity of polymer chains in case of the NaAlg–CH_3_COONa system. Thus, the anion type and its interaction with functional groups will affect the structural relaxation.

### 2.4 Thermogravimetric Analysis

Thermogravimetric analysis (TGA) has been performed to understand the effect of anion type on the thermal stability of polymer electrolytes since the thermal stability of electrolytes determines the operating temperature window of the energy storage system. Thermogram of sodium alginate (Figure S7) exhibits initial weight loss due to dehydration and second major weight loss in the temperature range of 203 and 251 °C due to decarboxylation of the carboxylate group and evolution of CO_2_,^57^ as well as fracture of glycosidic bonds, resulting in the fragmentation of NaAlg into monomeric units.^58,59^ The dependency of second weight loss on the dopant concentration can be observed in (Figures S7 and S8), with opposite nature in two electrolyte systems. The third minor weight loss observed from TG and DTG in both systems is related to anion decomposition. More significant losses were observed as the amount of salt increased. Thermal decomposition of the perchlorate anion can be represented as 2ClO_4_ → Cl_2_↑ + 4O_2_↑, and the acetate ion undergoes decarboxylation (CO_2_↑).

The Coats-Redfern integral eq 1 was applied to TG data to calculate the activation energy of the thermal degradation (main degradation) process of polymer electrolytes due to a single heating rate.^60^
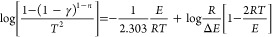
1in eq 1, *R* is the universal gas constant, *E* is the degradation activation energy, *n* is the order of the reaction, *T* is the absolute temperature, and γ is the fraction of sample decomposed at time *t* (at a given temperature). The γ was calculated using eq 2
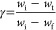
2

**Figure 6:**
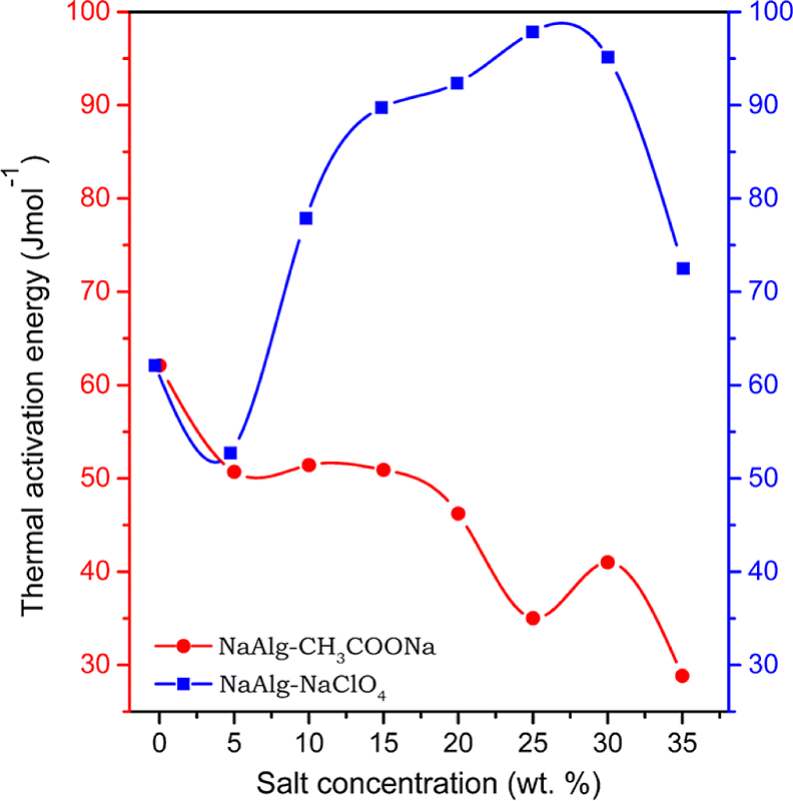
Variation of thermal activation energy with salt concentration for the NaAlg–CH_3_COONa and NaAlg–NaClO_4_ electrolyte system as per the Coats–Redfern integral equation.

In eq 2, *w*_i_, *w*_f_, and *w*_t_ are the initial weight, final weight, and weight at the given temperature of the sample, respectively. The activation energy for the second main degradation (between 203 and 251 °C) was calculated from the slope of the plot  against  using the relation *E* = 2.303*R* × slope, which results in a straight line for *n* = 1 (assuming first-order reaction), as shown in (Figures S9 and S10). High activation energy indicates a large amount of heat energy required to perturb the system with good thermal stability.^61^ A decrease in thermal activation energy for the NaAlg–CH_3_COONa electrolyte system indicates a reduction in thermal stability and vice versa in the case of the NaAlg–NaClO_4_ system and is due to the strong interaction of the ClO_4_^–^anion with the polymer matrix, as observed in Figure 6. The microstructural modification brought about in the matrix by the interaction of ions with the functional group is justified by the variation of the thermal activation energy with the salt content. The uneven variation of the activation energy is justified by the asymmetrical variation in the degree of crystallinity as well as *T*_g_. In both systems, the Na^+^ cation has interacted with the oxygen of COO^–^, the C–O–C of glycosidic linkage, and the –OH group. Since the variation of thermal activation energy is diametrically opposite in both systems, as observed in Figure 6, and thus, the type of anion and its interaction with the polymer host have a greater influence on the thermal activation energy/thermal stability in comparison with that of the cation.

### 2.5 Scanning Electron Microscopy Analysis

**Figure 7:**
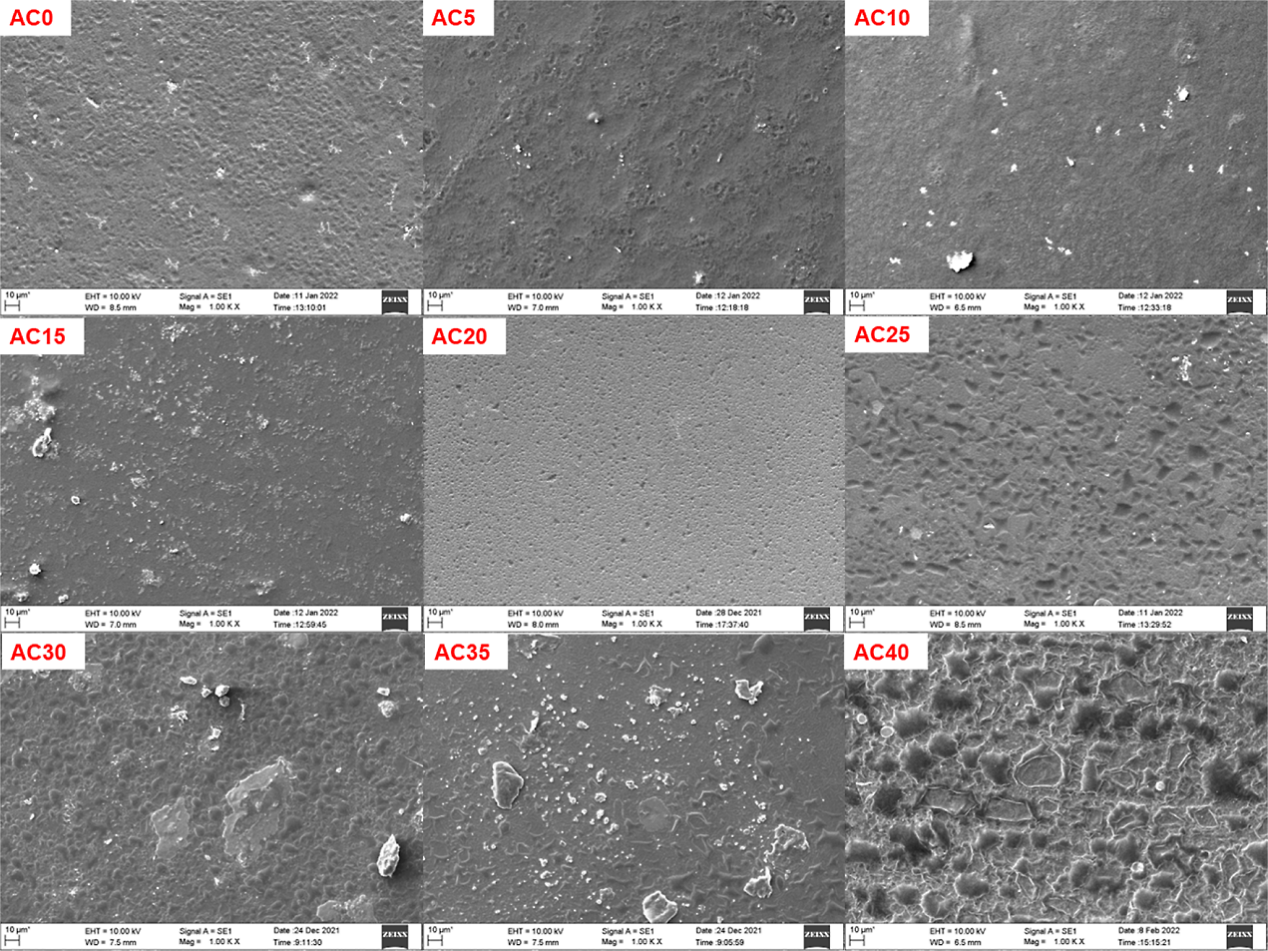
SEM micrographs of the NaAlg–CH_3_COONa complexed system.

**Figure 8:**
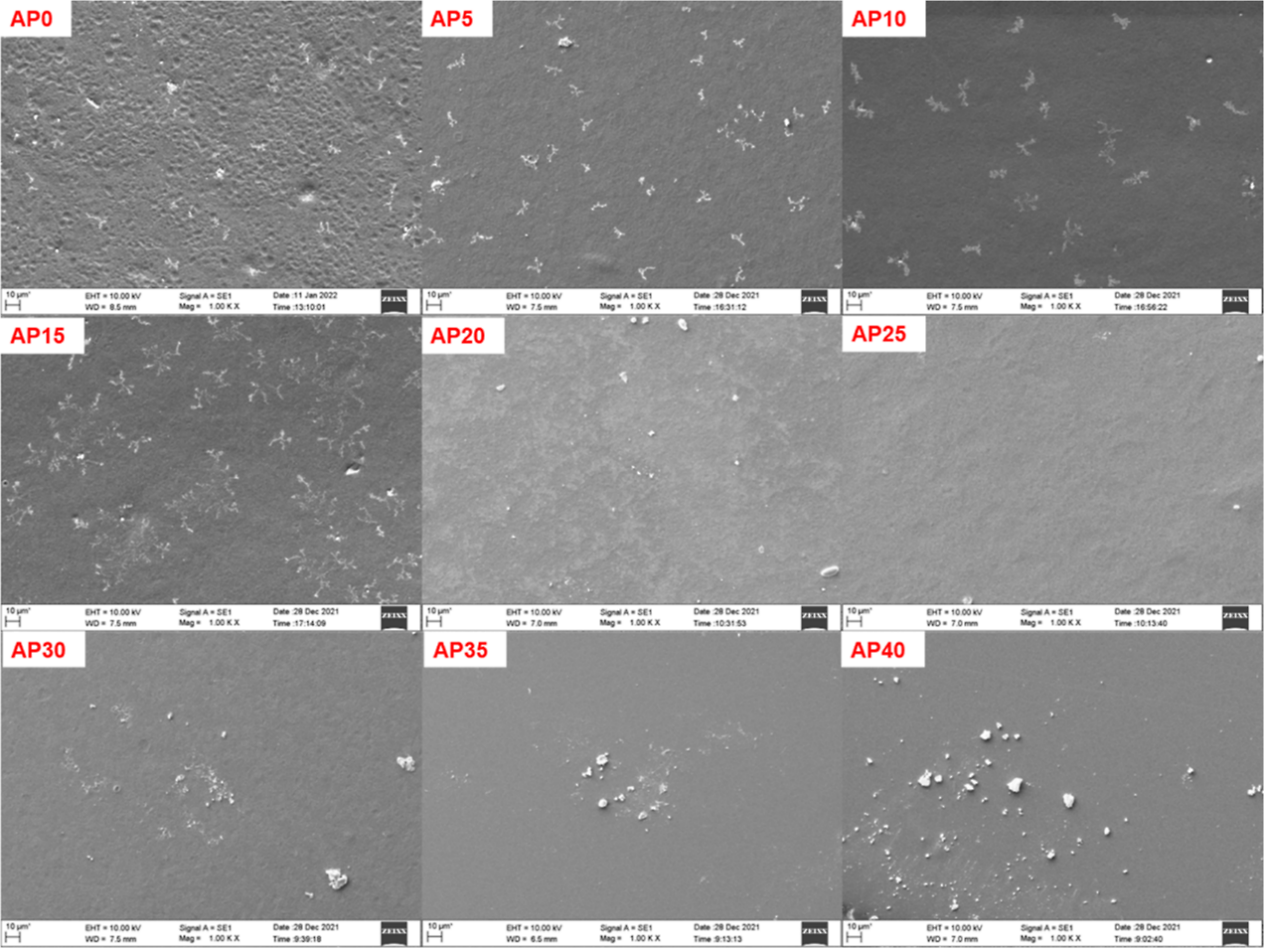
SEM micrographs of the NaAlg–NaClO_4_ complexed system.

The surface morphology of the polymer electrolyte films determines the texture of the electrolyte, has an influence on the electrolyte/electrode interface, and hence determines the ion migration at the interface and overall ionic conductivity, especially in the space charge polarization region. The membrane surface is influenced by the type of salt and its concentration as per the scanning electron microscopy (SEM) micrograph in both systems, as shown in Figures 7 and 8. Crystalline peaks of the salts are not observed in the XRD pattern, so there is no phase separation between the polymer and salt. The white spots observed on the micrographs are not related to undissociated salt since they are also observed in the pristine polymer. The variation in surface morphology can be related to the crystallinity.^62^ The appearance of the white spots (white cluster) suggests crystalline domains. In the NaAlg–CH_3_COONa electrolyte system, no white spots are observed for the AC20 sample that exhibits the lowest crystallinity. In the NaAlg–NaClO_4_ system, the AP35 sample exhibited minimal crystallinity, and therefore, its SEM image illustrated the smooth surface in the background with a minimal white cluster compared to others.

### 2.6 Mechanical Properties

**Figure 9:**
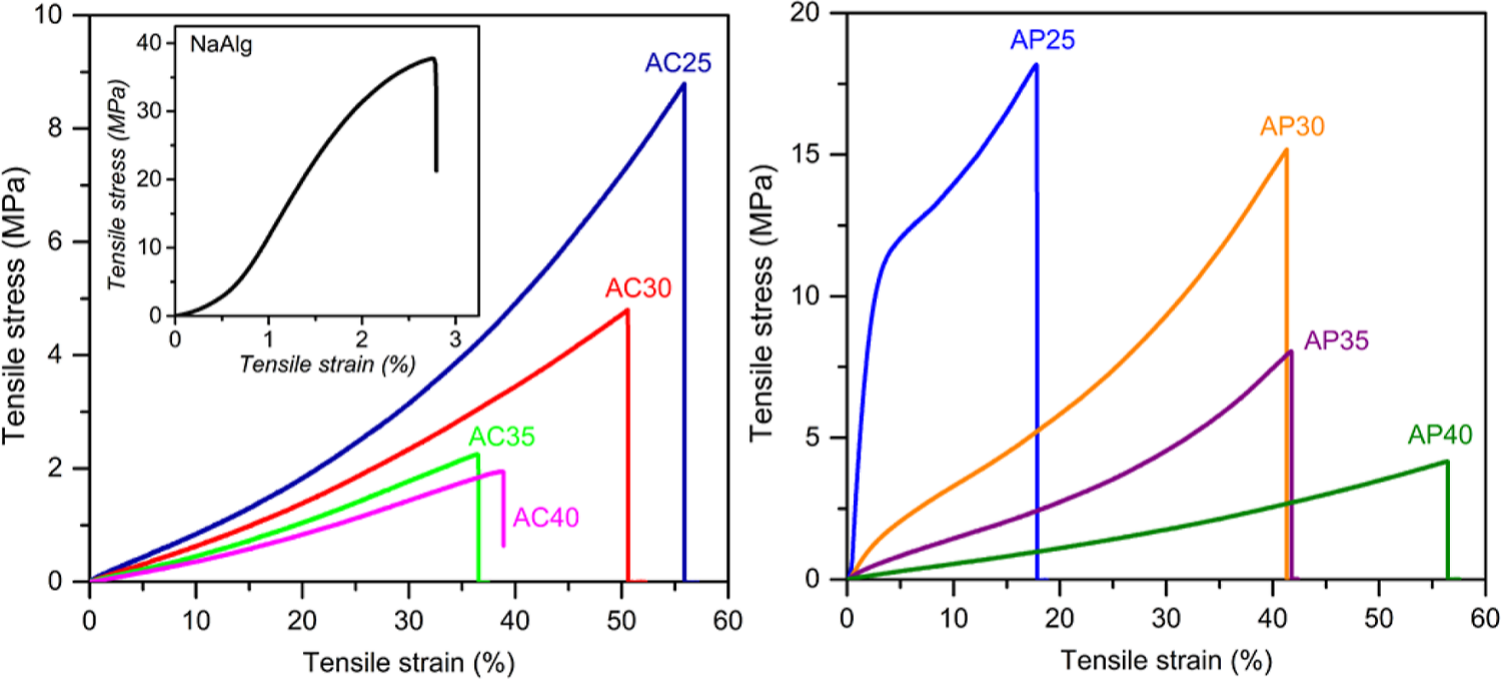
Stress–strain curves of NaAlg, 25, 30, 35, and 40 wt % of CH_3_COONa, and NaClO_4_ salt in the NaAlg polymer matrix.

Figure 9 shows the stress–strain curve of NaAlg, NaAlg–NaClO_4_, and NaAlg–CH_3_COONa electrolyte systems at room temperature. Young’s modulus and ultimate tensile strength (TS) were estimated from the curve and are summarized in Table S2. The mechanical strength of solid polymer electrolytes is an essential parameter when considering their large-scale application and improvising the energy density and cycle performance in energy storage applications. During the charge/discharge cycle of the battery, the volume of the electrode material changes, and the SPE in contact with the electrode needs to deform elastically with respect to the change in the electrode volume. Therefore, the need to have a suitable mechanical modulus (low elastic modulus) is important. According to Yue et al.,^63^ a mechanical strength of 30 MPa is optimized as the minimum requirement for the SPE to be incorporated in a lithium battery. Fan et al.^64^ reported that a mechanical strength in the order of 10^6^ Pa is sufficient to hinder Li-dendrite growth. NaAlg exhibited elongation at break <3%, exhibiting its brittle nature with low flexibility. In Figure 9, the downward shift in the curve in both cases with increasing salt content corresponds to a decrease in tensile stiffness and strength caused by the salt incorporated acting as a plasticizer. Modification in the molecular structure of the host by the formation of new inter/intramolecular interactions by the salt has resulted in variation in the mechanical properties. The nature of the curve of the NaAlg–CH_3_COONa system is slightly different from that of the NaAlg–NaClO_4_ system. The NaAlg–CH_3_COONa electrolyte system exhibits a marginally high elastomer (rubber-like behavior) nature compared with the NaAlg–NaClO_4_ electrolyte system. A correlation between the crystallization process and the mechanical properties is not possible in this case. The sufficient mechanical strength necessary for the incorporation of SPE in an energy storage system is not well addressed, and one can come to the conclusion that the necessary elastic modulus that needs to be possessed by the SPE is by carrying out the charge/discharge cycles.

### 2.7 Primary Battery

**Figure 10:**
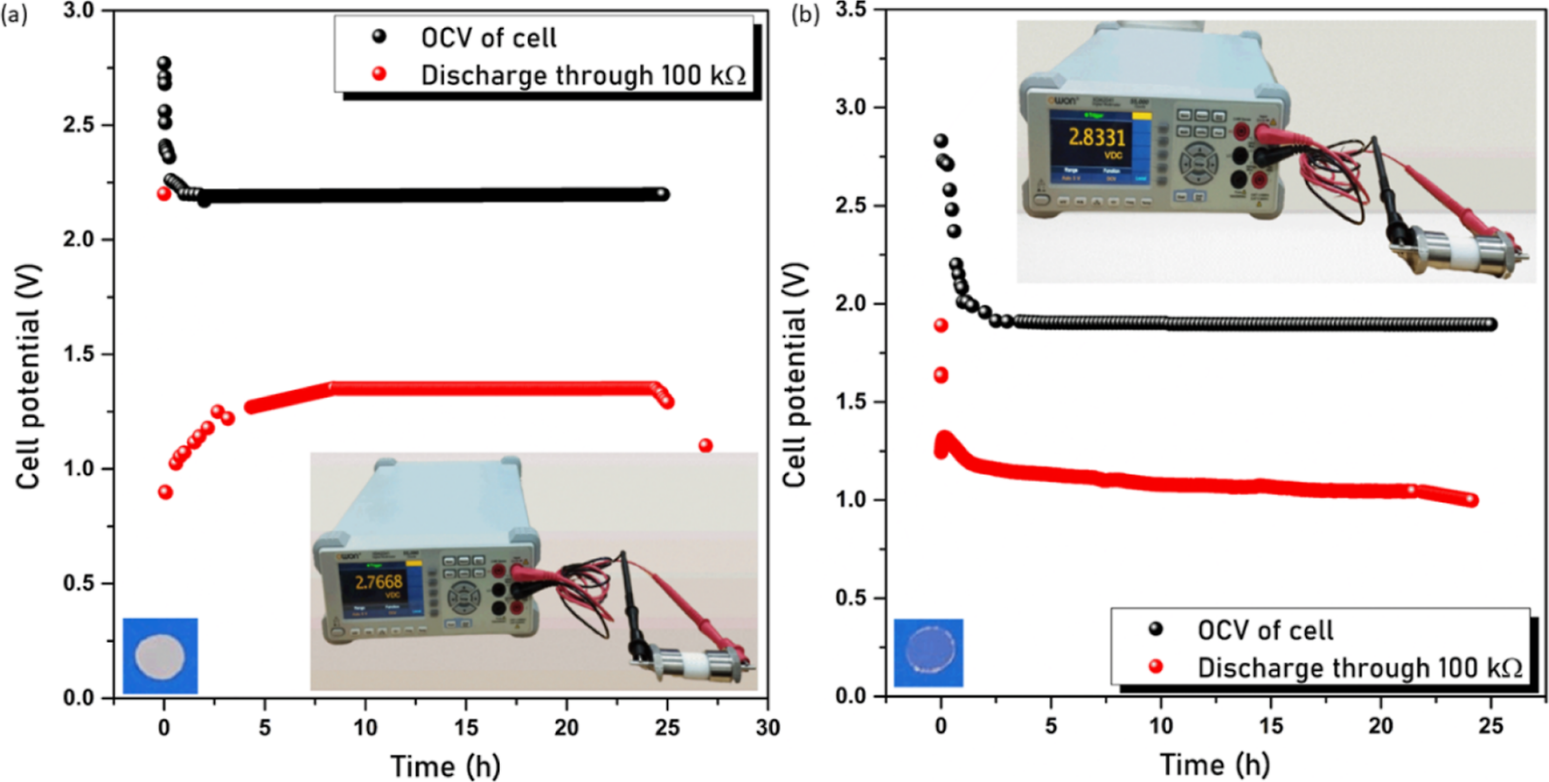
(a). Discharge curve of cell Na |AC40|I_2_ (3) + C (1) + AC40 (1) and (b) discharge curve of cell Na |AP40|I_2_ (3) + C (1) + AP40 (1) (inset displays photo image of the OCV and electrolyte samples).

The highest conducting samples, i.e., AC40 and AP40 with conductivity of about 10^–4^ S cm^–1^ and 10^–5^ S cm^–1^, respectively, are incorporated as the electrolyte in the dry cell in the configuration Na |Electrolyte| Cathode in a SWAGELOK CELL. The cathode consists of iodine (I_2_), graphite (C), and electrolyte (AC40/AP40) in the ratio 3:1:1, respectively. Iodine is used as the active material, and graphite is used for electron conduction purposes. Electrolyte (AC40/AP40) is added to them to reduce the interfacial resistance at the electrode/electrolyte interface.^65^ The mixture is prepared by grinding them using a pestle and mortar for a few hours and then making a pellet of diameter 13 mm under 5 tons of pressure using a pellet pressing machine. Na metal of diameter 12 mm and thickness 2 mm is incorporated as an anode. For comparison purposes, electrode materials are kept the same. The characteristics of Na |AC40| (I_2_ + C + AC40) and Na |AP40| (I_2_ + C + AP40) cells are shown in Figure 10a,b, respectively. The open circuit voltage (OCV) of 2.76 and 2.83 V was observed, shown as an inset in the graph. The initial drop in OCV is due to the formation of a passivating layer.^66^ Once the constant OCV is attained, the cell is subjected to discharge through a load. The cell comprising the AC40 sample exhibits energy density and power density of 260 mW h kg^–1^ and 16 mW kg^–1^ and the cell containing the AP40 sample displays energy density and power density of 178 mW h kg^–1^ and 9 mW kg^–1^, respectively. The difference in cell parameters as observed from Table S3 is due to the easy migration of ions within the electrolyte system because the ionic conductivity of the AC40 system is nearly one order greater than AP40. The results of the current study are in good agreement with those of past studies, as depicted in Table S4.

## 3 Conclusions

An insight into the role of anion size on the microstructure properties of solid polymer electrolytes based on a sodium alginate polymer is summarized. The availability of sufficient functional groups in NaAlg has assisted in salt dissociation up to 40 wt % of salt in the matrix, which proved to be a good candidate as a polymer host. The type of anion will influence the crystallinity of the polymer electrolyte based on its interaction with the polymer chain; the ClO_4_^–^ anion brought about changes in the crystalline phase and CH_3_COO^–^ in the amorphous phase of NaAlg. Polymer chain flexibility in the solid polymer electrolyte is decided by anion size and its charge delocalization, as per the DSC result. The ClO_4_^–^-based electrolyte is advantageous for wide-temperature-range application in comparison with that of the CH_3_COO^–^-based electrolyte since the thermal activation energy for the NaAlg ClO_4_^–^ electrolyte system is higher than that of the NaAlg–CH_3_COO^–^ electrolyte system, as per TGA/DTG studies. The type of salt influences the surface morphology as per the SEM micrograph, and its impact on ionic conductivity needs to be addressed. The NaAlg–CH_3_COONa electrolyte system was observed to have a soft texture when compared with the NaAlg–NaClO_4_ system, thus influencing the compatibility at the electrode–electrolyte interface. Anion size and its type have an influence on the crystallinity, glass transition temperature, and thermal stability of the polymer electrolyte. Further investigation on the impact of anion size on ionic conductivity and electrochemical properties will be accomplished in future work. A successful illustration of the highest-conducting samples, AC40 and AP40, in the dry cell is illustrated.

## 4 Materials and Methods

Sodium alginate (NaAlg) was purchased from S. D. FINE-Chem. Pvt. Ltd., Mumbai, India. Sodium perchlorate (NaClO_4_) and sodium acetate (CH_3_COONa) were procured from Sigma-Aldrich (USA) and Loba-Chemie (India), respectively. Cathode materials: Iodine (I_2_, MW = 253.81 g/mol) is supplied by Loba Chemie Pvt Ltd., and graphite (C, 100 μm, MW-12.01 g/mol) was purchased from S. D. Fine-Chem Pvt Ltd. All materials were used as received.

NaAlg:NaClO_4_and NaAlg:CH_3_COONa electrolyte films were prepared by a solution casting technique. The appropriate amounts of the NaAlg polymer and sodium salts, as in Table S5, were dissolved in deionized water and stirred continuously at 40 °C for 12 h until a clear, homogeneous mixture was obtained. Then, the mixed solution was poured onto a glass Petri dish and left to dry at room temperature for 7 days. The sample was then placed in a vacuum oven and dried at 100 °C for 5 h to further remove the trapped solvent. Film preparation was carried out only with up to 40 wt % salt in the polymer matrix. Otherwise, it will be considered a polymer-in-salt system if further salt is added to the matrix (polymer fraction is a minority compared to the salt). The compositions of the samples and their designations are tabulated in Table S5. The thickness of the films varied from 0.1 to 0.27 mm measured using a Mitutoyo micrometer.

### 4.1 FTIR Spectroscopy

FTIR spectroscopy of the films was recorded in the wavenumber region between 4000 and 400 cm^–1^ with a resolution of 4 cm^–1^ using IRPrestige-21 FTIR SHIMADZU to understand the interaction between the ions and functional groups.

### 4.2 Raman Spectroscopy

The transport parameters of the NaAlg:NaClO_4_ system were evaluated using the Raman deconvolution technique based on Raman spectra recorded at RT using a Lab RAM HR spectrometer (HORIBA, France) excited by a 532 nm Nd: YAG laser of output 100 mW.

### 4.3 X-Ray Diffraction

To understand the influence of anion type on the microstructure properties, especially on the crystallinity, XRD patterns were recorded using a third-generation Empyrean, Malvern Panalytical, The Netherlands with Cu Kα (λ = 0.154 nm) in the 2θ range between 5 and 90°.

### 4.4 Differential Scanning Calorimetry

The effect of anion size on the glass transition temperature (*T*_g_) of the electrolyte was investigated by the DSC technique. DSC traces were registered on a NETZSCH DSC 204F1 Phoenix differential analyzer in the temperature range of 20 and 200 °C at a scan rate of 10 °C min^–1^ under nitrogen gas circulation at a flow rate of 60 mL min^–1^. Samples weighing 1 to 3 mg were placed in a concavus Al crucible with a pierced lid.

### 4.5 Thermogravimetric Analysis

TGA was carried out to determine the thermal stability of the prepared SPE. The measurement was performed using an SDT Q600 V20.9 build 20 instrument. A sample of approximately 5 mg of weight was placed on a platinum crucible. The platinum crucible was inserted into the enclosed machine chamber and heated at a scan rate of 10 °C min^–1^ from 20 to 500 °C with nitrogen as a purge gas flowing at 80 mLmin^–1^.

### 4.6 Scanning Electron Microscope

The influence of the salt type on the surface morphology of the films was studied using the SEM image. To improve the imaging, the samples were coated with gold before recording. SEM images were recorded by a Zeiss EVO 18 SEM at an accelerating voltage of 10 kV.

### 4.7 Dynamic Mechanical Analysis

To check the sufficient mechanical strength required for the incorporation of SPE in energy storage systems, the tensile strength and modulus of elasticity were tested using a model Instron-3366 universal testing machine according to the ASTM standard method D882.^67^ Specimens were cut in a rectangular strip (length 100 mm, width 25 mm) for testing with initial grip separation fixed at 50 mm and tested at a cross-head speed of 5 mm min^–1^. Prior to analysis, samples were placed in a desiccator containing a saturated solution of NaBr at a relative humidity of 55% for 24 h.

### 4.8 Primary Battery

The open-circuit potential and discharge characteristics of the cell are studied using an OWON XDM2041 digital multimeter instrument.

## Supporting Information Available

Full synthetic procedure and characterization (FTIR, XRD, DSC, TGA, and UTM) and data extraction from original plots (PDF)

## Notes

Data will be made available on request.

## Notes

Ethics approval and consent to participate: This article does not contain any studies with human participants or animals performed by any of the authors.

## Notes

Consent for publication: The authors hereby consent to publication of the present research work in this journal, if selected for publication.
