# Supplementary material for: Deciphering the Effect of Microstructural Modification in Sodium Alginate-Based Solid Polymer Electrolyte by Unlike Anions
Source: ACS Omega. 2023 Nov 13;8(46):43632–43. doi: 10.1021/acsomega.3c05094 (PMC10683634; doi:10.1021/acsomega.3c05094)
Supplement: Supplementary file 1 — ao3c05094_si_001.pdf [file ao3c05094_si_001.pdf]

Supporting information for

Deciphering the Effect of Microstructural Modification in Sodium Alginate based Solid Polymer Electrolyte by Unlike Anions

Supriya K Shetty[a], Ismayil[a*], Ikhwan Syafiq Mohd Noor[b], Sudhakar Narahari Yethadka[c], Pradeep Nayak[a]

[a]Department of Physics, Manipal Institute of Technology, Manipal Academy of Higher Education, Manipal 576104, Karnataka, India

[b]Physics Division, Centre of Foundation Studies for Agricultural Science, Universiti Putra Malaysia, 43400 Serdang, Selangor Darul Ehsan, Malaysia

[c]Department of Chemistry, Manipal Institute of Technology, Manipal Academy of Higher Education, Manipal 576104, Karnataka, India

[*]*Corresponding Author e-mail*: ismayil.mit@manipal.edu, ismayil.486@gmail.com,
Tel: +91 98454 97546

Supplementary Data

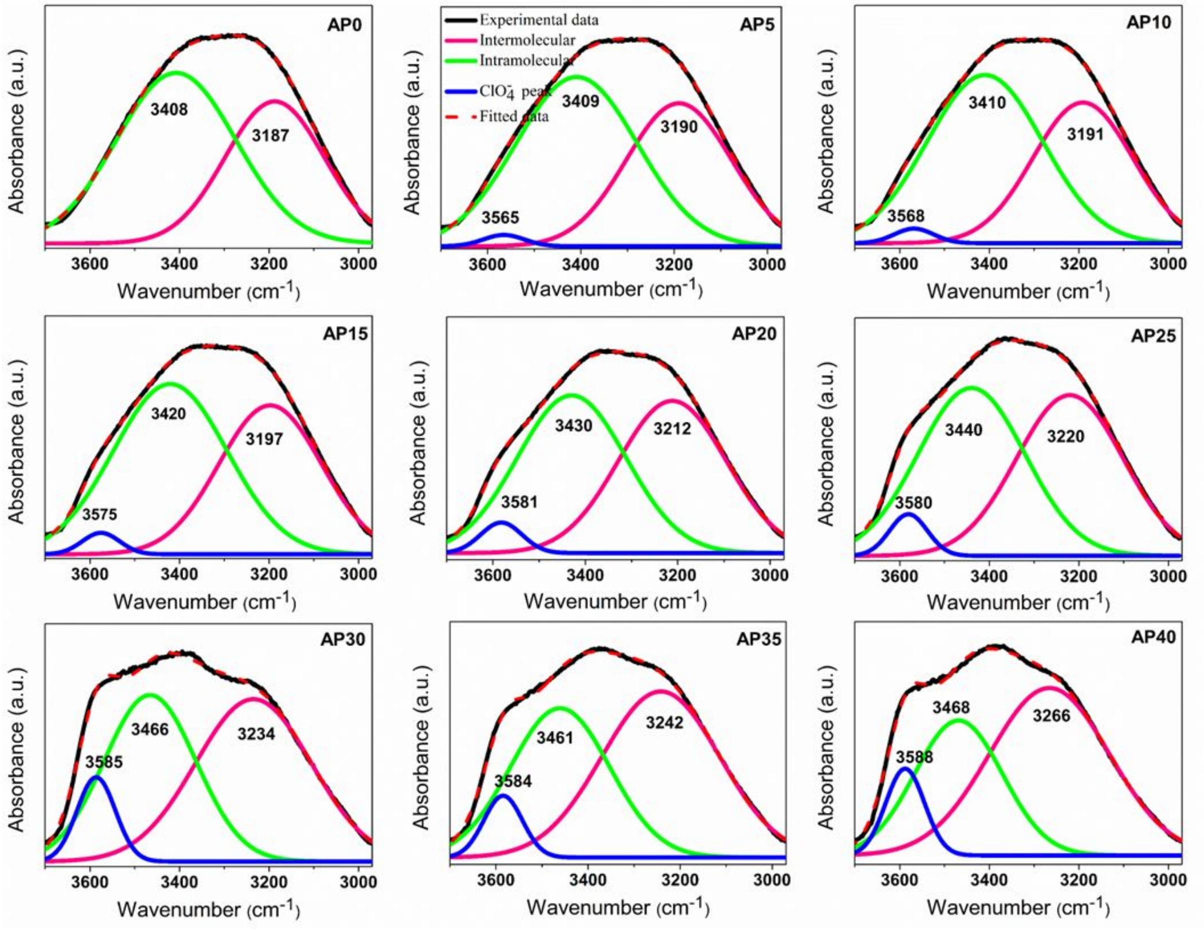

Figure S1. FTIR deconvolution of $NaAlg - NaClO_4$ system in –OH region.

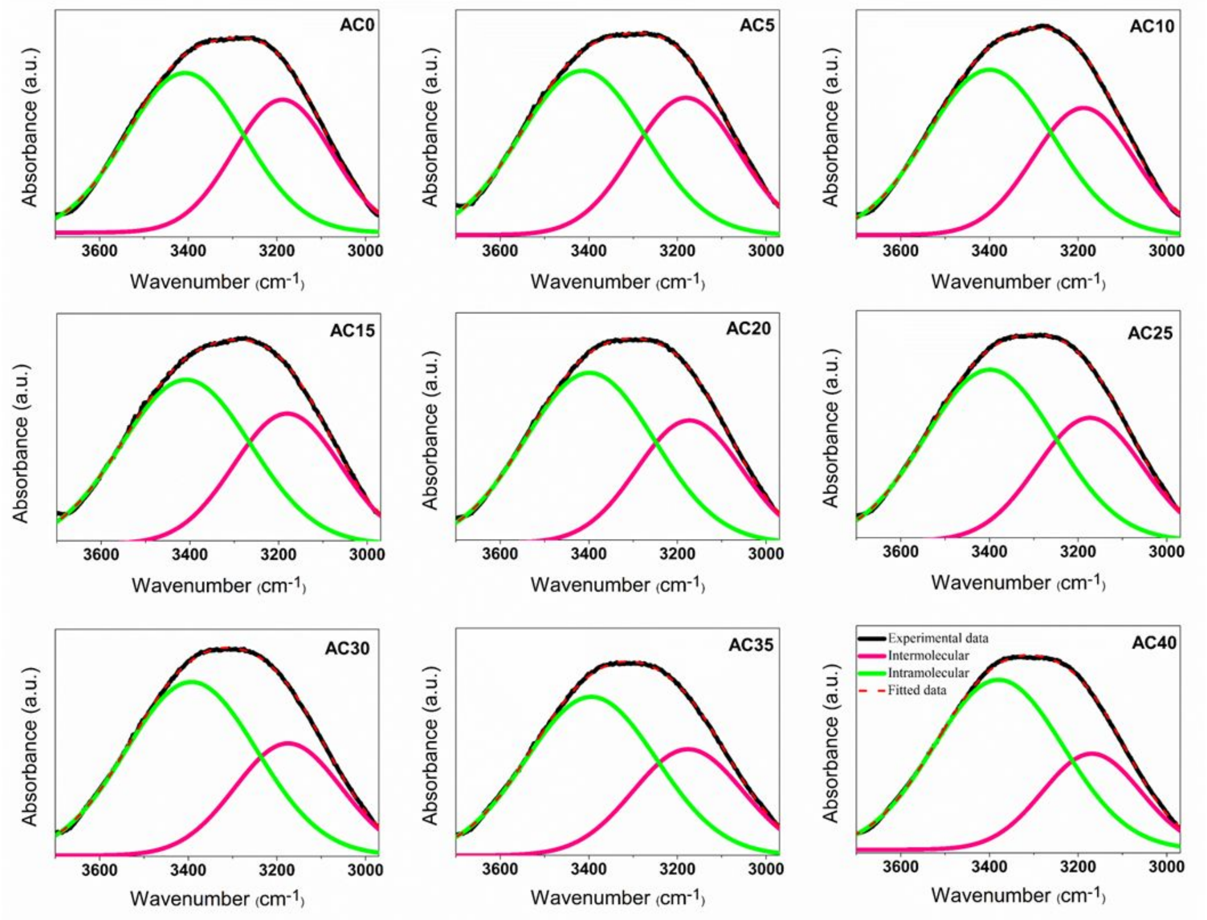

Figure S2. FTIR deconvolution of $NaAlg - CH_3COONa$ system in –OH region.

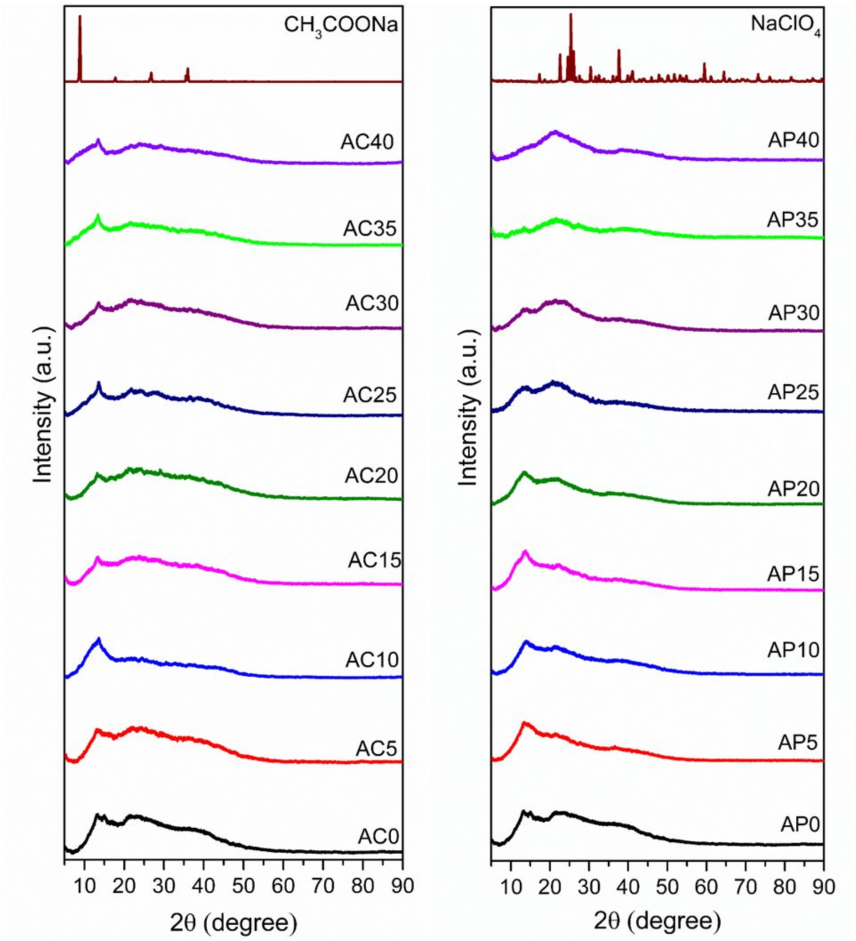

Figure S3. XRD pattern of $NaAlg$, $NaClO_4$, CH_3COONa, $NaAlg-NaClO_4$, $NaAlg-CH_3COONa$ polymer electrolyte system.

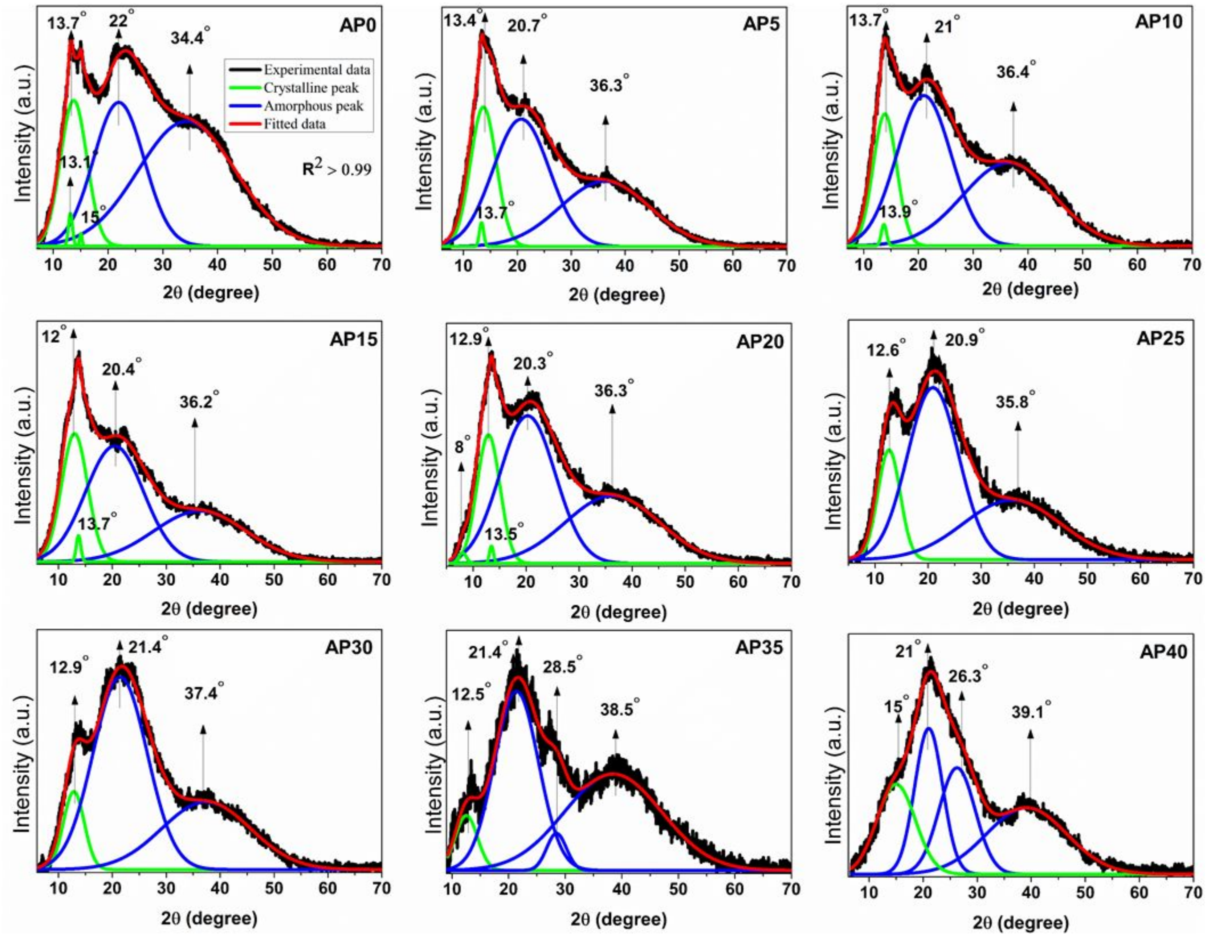

Figure S4. Deconvoluted XRD pattern of $NaAlg - NaClO_4$ solid polymer electrolyte system.

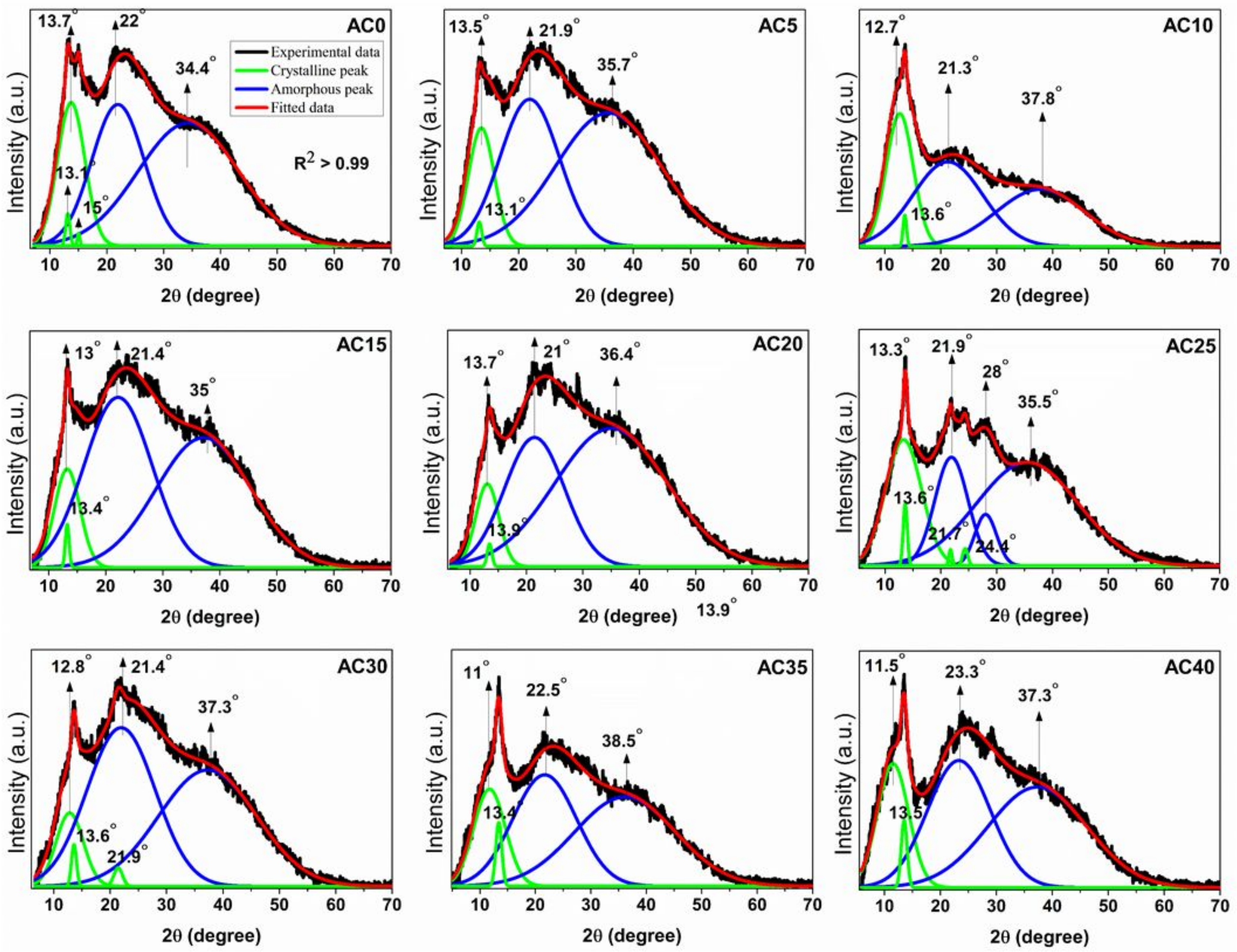

Figure S5. Deconvoluted XRD pattern of $NaAlg - CH_3COONa$ solid polymer electrolyte system.

Table S1. Variation in crystallinity (χ_c) with salt concentration for $NaAlg - CH_3COONa$ & $NaAlg - NaClO_4$ electrolyte system.

Sample	Crystallinity (χ_c) %	Sample	Crystallinity (χ_c) %
AC0	17.09	AP0	17.09
AC5	12.23	AP5	22.10
AC10	25.11	AP10	16.91
AC15	10.08	AP15	23.24
AC20	7.98	AP20	17.57
AC25	24.60	AP25	14.38
AC30	9.65	AP30	8.99
AC35	16.00	AP35	6.37

AC40	19.17	AP40	20.07

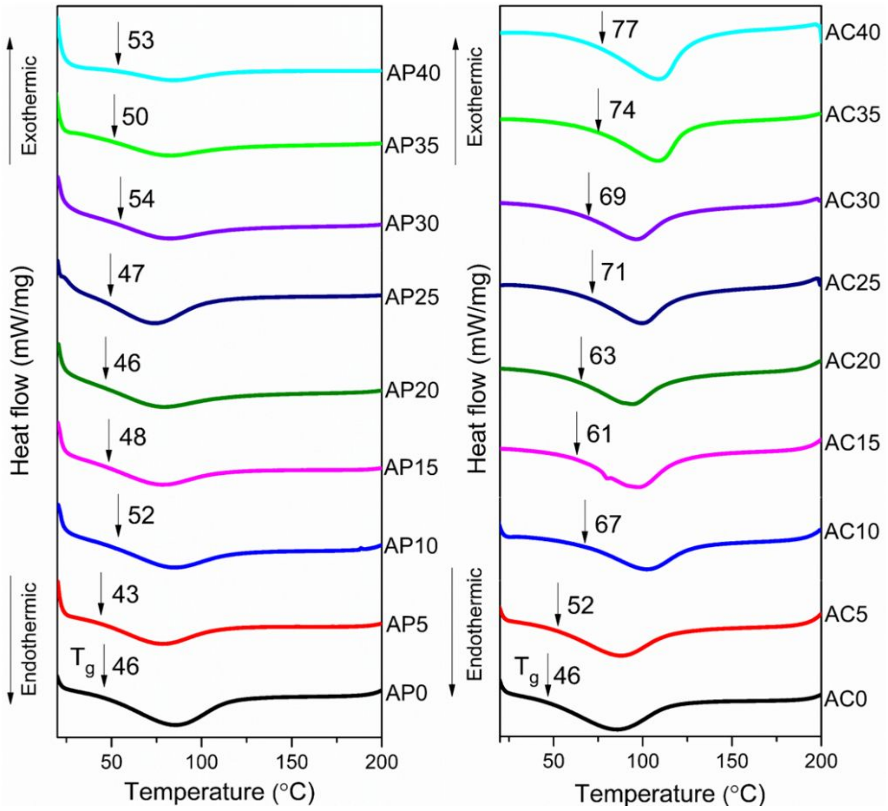

Figure S6. DSC thermograms of $NaAlg - NaClO_4$ and $NaAlg - CH_3COONa$ electrolyte system.

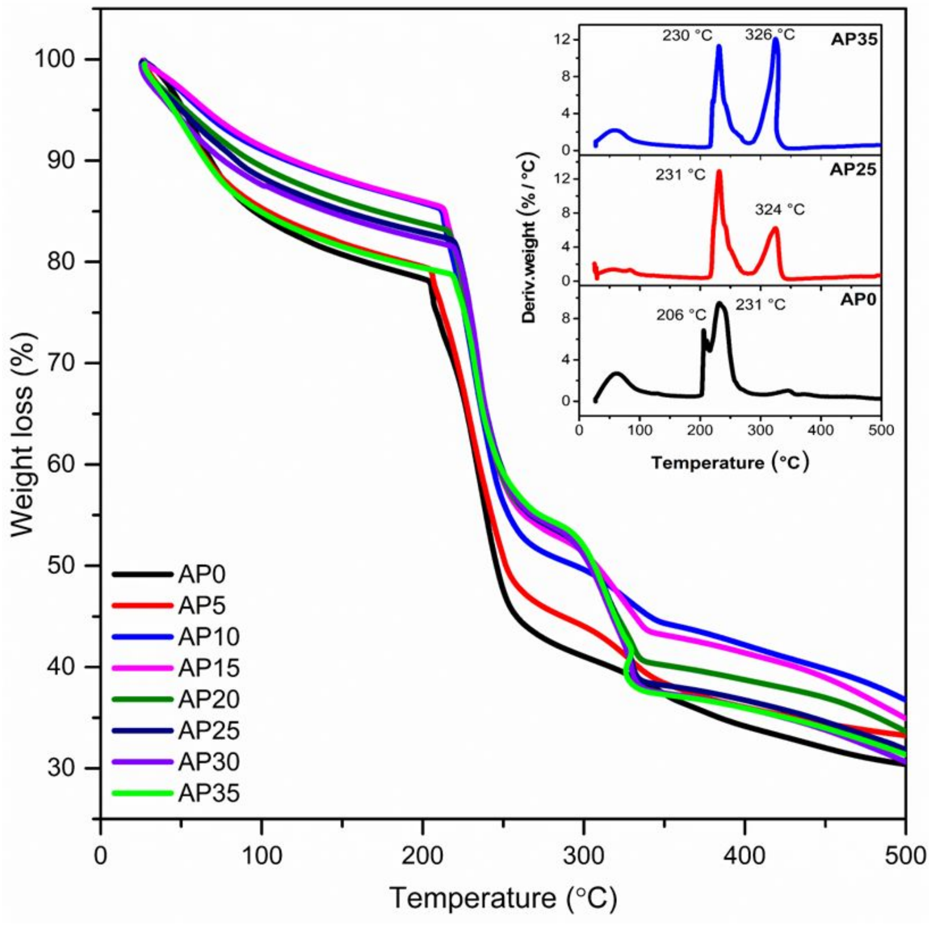

Figure S7. TGA/DTG plot of $NaAlg$ and $NaAlg - NaClO_4$ electrolyte system.

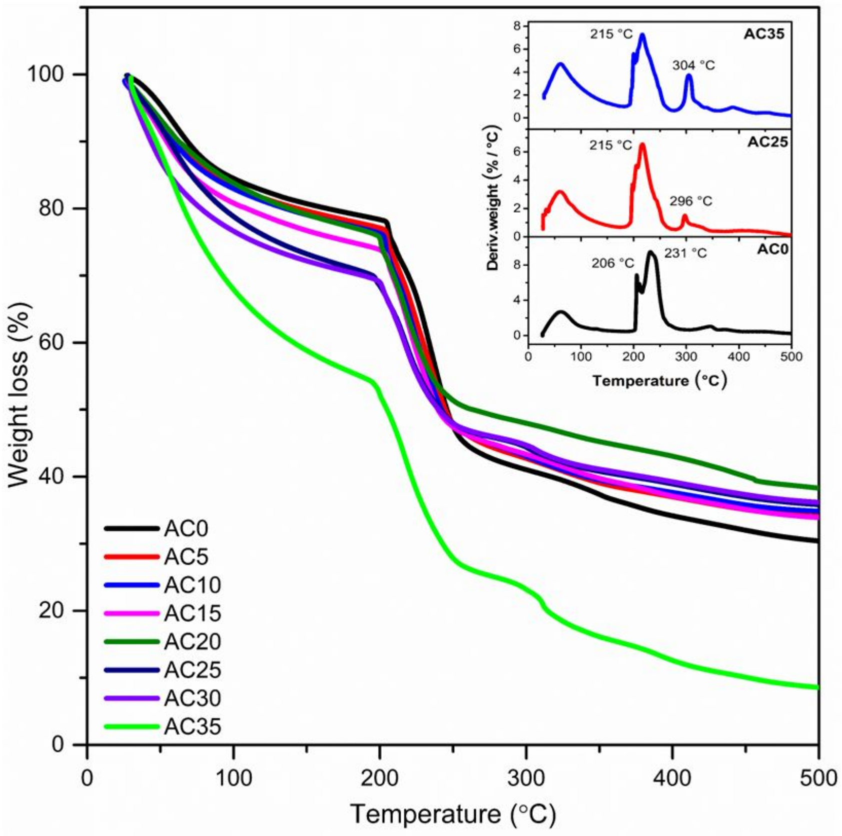

Figure S8. TGA/DTG plot of $NaAlg$ and $NaAlg-CH_3COONa$ of electrolyte system.

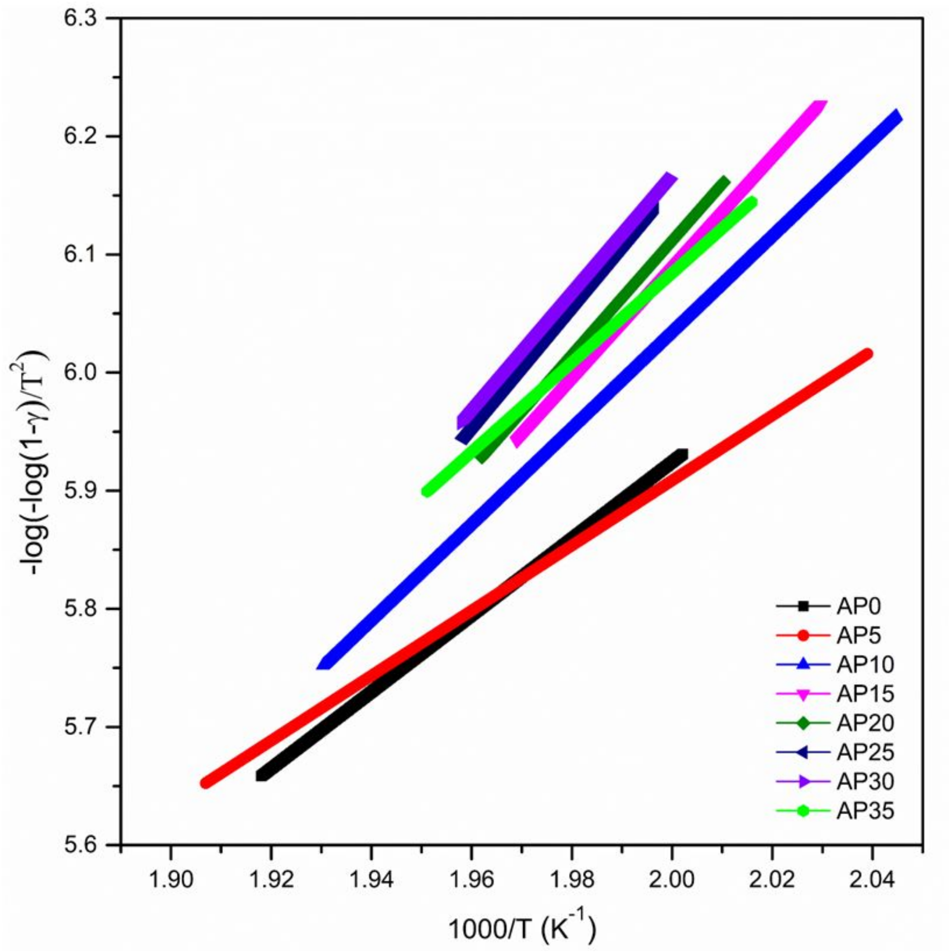

Figure S9. Variation of $-log\left[\frac{-log\,(1-\gamma)}{T^2}\right]$ vs $\frac{1000}{T}$ for $NaAlg - NaClO_4$ electrolyte system.

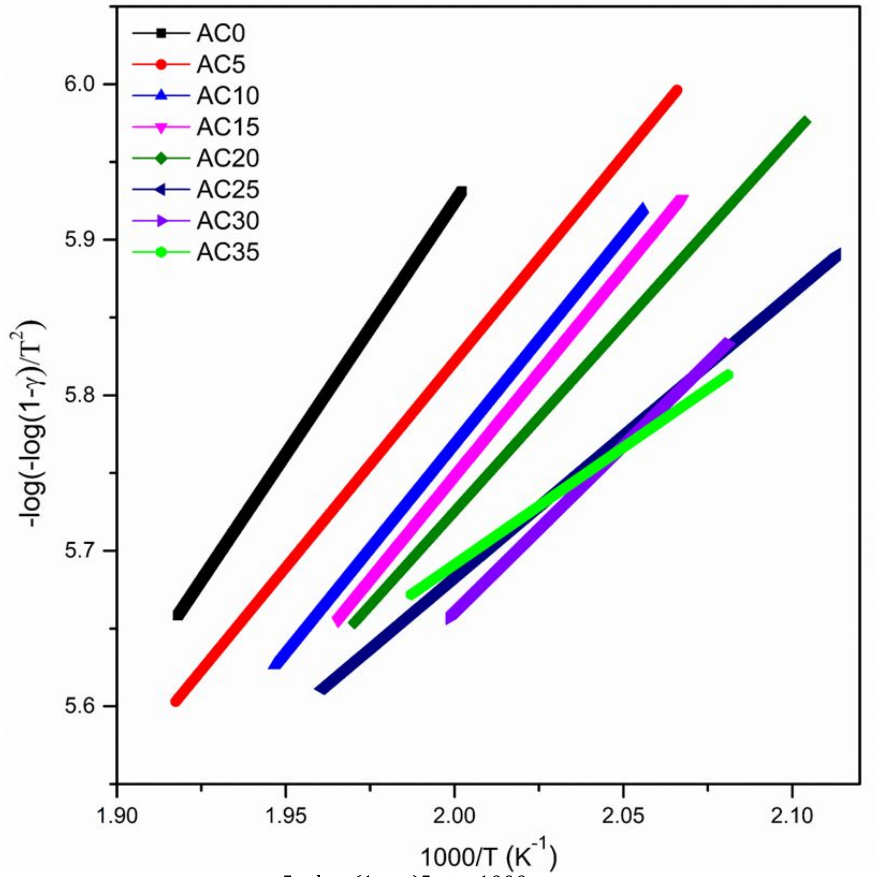

Figure S10. Variation of $-log\left[\frac{-log\,(1-\gamma)}{T^2}\right]$ vs $\frac{1000}{T}$ for $NaAlg - CH_3COONa$ electrolyte system.

Table S2. Mechanical properties of solid polymer electrolyte films.

Sample	Tensile strength (MPa)	Young's modulus (MPa)	Sample	Tensile strength (MPa)	Young's modulus (MPa)
**AC0**	37.75	2304.81	**AP0**	37.75	2304.81
**AC25**	8.79	27.33	**AP25**	18.18	614.30
**AC30**	4.80	13.25	**AP30**	15.18	57.62
**AC35**	2.25	7.60	**AP35**	8.05	35.69
**AC40**	1.95	6.39	**AP40**	4.17	10.60

Table S3. Comparison of cell parameters of two systems.

Cell parameters	Measured Values	
	Na |AC40| (I_2 + C + AC40)	Na |AP40| (I_2 + C + AP40)
Cell area (cm^2)	1.23	1.23
Weight of the cell (g)	0.82	1.10
Effective diameter of the cell (cm)	1.30	1.30
Thickness of the cell(cm)	0.4	0.42
Open circuit voltage (V)	2.76	2.83
Current drawn (μA)	10	9.8
Current density (μAcm^{-2})	8.13	7.96
Discharge time plateau region (h)	16	20
Discharge capacity (μAh^{-1})	0.625	0.49
Energy density ($mWhkg^{-1}$)	260	178
Power density ($mWkg^{-1}$)	16	9

Table S4: Comparison of the OCV achieved in the current study with those from earlier research utilizing an anode made of sodium.

Electrolyte	Cathode	OCV	Reference
Gellan gum + $NaClO_4$	MnO_2+C	2.99	[1]
Poly(vinyl pyrrolidone) + NaI	I_2+C+Electrolyte	2.98	[2]
Poly(vinyl pyrrolidone) + $NaNO_3$	I_2+C+Electrolyte	2.65	[3]
Poly(ethylene oxide) + $NaYF_4$	I_2+C+Electrolyte	2.45	[4]
NaAlg + CH_3COONa	I_2+C+Electrolyte	2.76	Present work
NaAlg + $NaClO_4$	I_2+C+Electrolyte	2.83	Present work

Table S5: Compositions and thickness of the samples

Composition ($NaAlg + NaClO_4$)		Label	Thickness (mm)	Composition ($NaAlg + CH_3COONa$)		Label	Thickness (mm)
$NaAlg$ (g)	$NaClO_4$ (g)			$NaAlg$ (g)	(mm)		
2.0	0	AP0	0.17	2.0	0	AC0	0.17
1.9	0.1	AP5	0.11	1.9	0.1	AC5	0.16
1.8	0.2	AP10	0.14	1.8	0.2	AP10	0.11
1.7	0.3	AP15	0.10	1.7	0.3	AC15	0.15
1.6	0.4	AP20	0.12	1.6	0.4	AC20	0.18
1.5	0.5	AP25	0.16	1.5	0.5	AC25	0.22
1.4	0.6	AP30	0.16	1.4	0.6	AC30	0.20
1.5	0.7	AP35	0.17	1.5	0.7	AC35	0.23
1.6	0.8	AP40	0.15	1.6	0.8	AC40	0.27

References

(1) Kani Ajay Babu, M.; Jayabalakrishnan, S. S.; Selvasekarapandian, S.; Aafrin Hazaana, S.; Meera Naachiyar, R.; Muniraj Vignesh, N. Development and Characterization of Biopolymer Electrolyte Based on Gellan Gum for the Fabrication of Solid-State Sodium-Ion Battery. *Ionics* **2023**. https://doi.org/10.1007/s11581-023-05210-9.

(2) Venkata Subba Rao, C.; Ravi, M.; Raja, V.; Balaji Bhargav, P.; Sharma, A. K.; Narasimha Rao, V. V. R. Preparation and Characterization of PVP-Based Polymer Electrolytes for Solid-State Battery Applications. *Iran Polym J* **2012**, *21* (8), 531–536. https://doi.org/10.1007/s13726-012-0058-6.

(3) Reddy, M. J.; Sreekanth, T.; Chandrashekar, M.; Rao, U. V. S. Ion Transport and Electrochemical Cell Characteristic Studies of a New (PVP 1 NaNO3) Polymer Electrolyte System. *Journal of Materials Science* **2000**, *35* (11), 2841–2845. https://doi.org/10.1023/A:1004707521250.

(4) Sreepathi Rao, S.; Jaipal Reddy, M.; Laxmi Narsaiah, E.; Subba Rao, U. V. Development of Electrochemical Cells Based on (PEO + NaYF4) and (PEO + KYF4) Polymer Electrolytes. *Materials Science and Engineering: B* **1995**, *33* (2), 173–177. https://doi.org/10.1016/0921-5107(94)01206-7.
